# Transposable elements generate population-specific insertional patterns and allelic variation in genes of wild emmer wheat (*Triticum turgidum ssp. dicoccoides*)

**DOI:** 10.1186/s12870-017-1134-z

**Published:** 2017-10-27

**Authors:** Katherine Domb, Danielle Keidar, Beery Yaakov, Vadim Khasdan, Khalil Kashkush

**Affiliations:** Department of Life Sciences, Ben-Gurion University, 84105 Beer-Sheva, Israel

**Keywords:** Copy number variation, *Dicoccoides*, Emmer wheat, Genetic variation, Transposable elements, TE dynamics

## Abstract

**Background:**

Natural populations of the tetraploid wild emmer wheat (genome AABB) were previously shown to demonstrate eco-geographically structured genetic and epigenetic diversity. Transposable elements (TEs) might make up a significant part of the genetic and epigenetic variation between individuals and populations because they comprise over 80% of the wild emmer wheat genome. In this study, we performed detailed analyses to assess the dynamics of transposable elements in 50 accessions of wild emmer wheat collected from 5 geographically isolated sites. The analyses included: the copy number variation of TEs among accessions in the five populations, population-unique insertional patterns, and the impact of population-unique/specific TE insertions on structure and expression of genes.

**Results:**

We assessed the copy numbers of 12 TE families using real-time quantitative PCR, and found significant copy number variation (CNV) in the 50 wild emmer wheat accessions, in a population-specific manner. In some cases, the CNV difference reached up to 6-fold. However, the CNV was TE-specific, namely some TE families showed higher copy numbers in one or more populations, and other TE families showed lower copy numbers in the same population(s).

Furthermore, we assessed the insertional patterns of 6 TE families using transposon display (TD), and observed significant population-specific insertional patterns. The polymorphism levels of TE-insertional patterns reached 92% among all wild emmer wheat accessions, in some cases. In addition, we observed population-specific/unique TE insertions, some of which were located within or close to protein-coding genes, creating allelic variations in a population-specific manner. We also showed that those genes are differentially expressed in wild emmer wheat.

**Conclusions:**

For the first time, this study shows that TEs proliferate in wild emmer wheat in a population-specific manner, creating new alleles of genes, which contribute to the divergent evolution of homeologous genes from the A and B subgenomes.

**Electronic supplementary material:**

The online version of this article (10.1186/s12870-017-1134-z) contains supplementary material, which is available to authorized users.

## Background

Wild emmer wheat, *Triticum turgidum ssp. dicoccoides*, is an allotetraploid annual self-pollinating grass species formed ~ 300,000–500,000 years ago, by hybridization between two diploid species harboring AA and BB genomes [1, 2]. Based on molecular evidence, it was proposed that the allopolyploidization event that led to the creation of wild emmer wheat occurred in Mt. Hermon and upper Jordan Valley area [3–5]. During its evolution, wild emmer wheat spread within the region, surviving several rapid climate changes. The last of which, known as “the big freeze”, was characterized by temperature drops and dry conditions, occurred ~11,000 years ago [6, 7]. Paleobotanical findings from this period suggest wild emmer wheat might have been distributed along the “Levantine Corridor”, the narrow strip between southeastern Turkey and the Sinai peninsula, at least in its central-southern part [8].

The present-day wild emmer wheat is distributed in a patchy manner throughout the middle east Fertile Crescent, in various environmental conditions differing in soil types, mean annual temperatures, rainfall levels and other abiotic and biotic conditions [4].

In Israel, wild emmer wheat can be found between Mt. Hermon in the north and Mt. Amasa (Judea desert) in the south, as over 20 isolated or semi-isolated populations. The ecogeographical conditions (rainfall, temperature, humidity) do not vary dramatically among populations, except for the marginal populations, e.g. the temperatures frequently dropping below 0 °C in the winter in Mt. Hermon, and very low rainfall levels in Mt. Amasa [4, 9]. Wild emmer wheat populations from Israel were tested for phenotypic differences, and high variability was found in traits such as vegetative weight, seed weight, flowering time, pathogen resistance [10], drought tolerance [11] and other parameters, many of them of high agronomic importance. Several studies that have investigated the genetic structure and differentiation of wild emmer wheat on both the macro- and micro-scale level were published using limited numbers of genetic markers [12–14]. Genetic variation within and between wild emmer wheat populations was tested previously using genetic markers allowing the examination of a limited number of sites [4, 12, 14, 15], and possible adaptive genetic differentiation was found.

The relatively large genome of wild emmer wheat (~12 Gbp) contains 80–90% transposable elements (TEs) from both main classes [16–18]: class I (retrotransposons or RNA elements) and class II (DNA elements). Retrotransposons are mobile elements that proliferate by creating exact copies of themselves via RNA intermediates. They are subdivided into retrotransposons with long terminal direct repeats (LTRs) at their termini, and non-LTR retrotransposons. DNA elements have terminal inverted repeats, and are further subdivided based on their TIR sequence, transposase sequence and target site duplication. Both TE classes include autonomous elements encoding all the enzymes required for transposition, and non-autonomous elements that rely on enzymes encoded by autonomous elements [19]. The autonomous LTR retrotransposons have given rise to non-autonomous LARDs (Large Retrotransposon Derivatives) and TRIM (Terminal Repeats in Miniature). *SINE* (Small Interspersed Nuclear Elements) retrotransposons, unlike other non-autonomous TEs, are not derivatives of autonomous elements, and have likely originated from retrotransposition of *PolIII* transcripts. TIRs DNA elements have given rise to multiple non-autonomous elements of various sizes, including the smallest (dozens to hundreds of bp in length) DNA elements designated MITEs (Miniature Inverted Repeat Transposable Elements) possessing TIRs and a short non-coding internal sequence [19]. TE proliferation and elimination are two major contributors to change in the genome sizes of flowering plants [20]. In addition to shaping the size of the genome, TEs may affect gene expression in various ways: (1) insertions into exons usually lead to loss of function or alter the gene product, (2) insertion into introns can cause new alternative splicing [21], and (3) insertion into regulatory sequences upstream or downstream to a gene may lead to either upregulation or downregulation, depending on the role of a particular regulatory sequence [22]. Furthermore, as a natural target for various epigenetic silencing mechanisms, TEs induce formation of heterochromatic islands, which may lead to down-regulation of genes located within flanking regions [22, 23].

In this study, we aimed to elucidate the dynamics of TEs in natural habitats of wild emmer wheat accessions. We analyzed in detail 12 TE families representing both TE classes in 50 accessions collected from five geographically isolates sites (populations). The analyses included: (1) Assessment of the variation in TE copy numbers in emmer wheat accessions; (2) Assessment of the insertional patterns of TEs within and between the five wild emmer wheat populations; (3) Identification of population-specific/unique TE insertions within protein-coding genes and assessment of the allelic variation caused by TEs; and (4) Assessment of the expression profile of genes that harbor polymorphic TE insertions in wild emmer wheat. To this end, we have observed significant copy number variations of TEs within and between wild emmer wheat populations. In addition, for the first time, we report on TE-derived allelic variation in protein-coding genes in a population-specific/unique matter in natural populations of emmer wheat. The impact of TEs on genetic diversity and evolution of wild emmer wheat is discussed.

## Methods

### Plant material

Wild emmer wheat (*T. turgidum* ssp. *dicoccoides*) accessions used in this study were gathered from five geographically isolated sites (populations) in Israel, and were provided to us by Israeli stock centers, as following: accessions from Mount Hermon, Tabgha and Jaba collecting sites, are available in the Wild Cereals gene bank (WCGB) – Institute of Evolution, University of Haifa; Mount Hermon, while accessions from Amiad and Mount Amasa are available in the Lieberman Germplasm Bank, Institute for Cereal Crops Improvement, Tel-Aviv University. For details on the exact locations of the collection sites and eco-geographical conditions, see Additional file 1: Table S1 in our recent publication [24]. Next, 10–20 accessions from each population were grown in a greenhouse under common garden conditions (25 °C, 16 h/8 h long day). Leaf material was harvested approximately 4 weeks post germination for DNA extraction using the DNeasy plant mini kit (Qiagen), and total RNA extraction using TriReagent (Sigma-Aldrich).

### Assessment of TE copy numbers using real-time quantitative PCR (qPCR)

The copy number variation of 8 DNA elements (class II) and 4 retrotransposons (class I) in 50 wild emmer wheat accessions was determined by real-time qPCR. Details on TE families analyzed in this study are shown in Table 1. TE-specific primers (Additional file 1: Table S1) were designed using Primer Express v3.0 software (Applied Biosystems). Quantitative PCR reactions using genomic DNA templates isolated from wild emmer wheat accessions were performed by a 7500 Fast Real-Time PCR system and analyzed using the 7500 version 2.0.5 software (Applied Biosystems). Each reaction contained: 7.5 μl KAPA SYBR FAST qPCR Master Mix, 0.3 μl ROX Low (KAPA BIOSYSTEMS), 1 μl forward and 1 μl reverse primers (10 μM), 0.2 μl ddH_2_O and 5 μl template genomic DNA (0.25 ng/μl). The PCR conditions for these reactions were: 95°c for 3 min, repeat (95°c for 1 min, 60°c for 1 min) 40 times; following the last cycle, a melting curve was performed in order to validate product specificity. For each DNA sample (accession) a qPCR reaction was performed using three replicates. Data for each sample was received as C_T_ – threshold cycle of the PCR amplification, normalized to the C_T_ of *VRN1*, a known single copy number gene [25] used as an endogenous control [26–28]. A comparative 2^-ΔΔCT^ method was then used to determine the relative quantity (RQ) of the target in each sample by comparing the normalized target quantity in each sample to the normalized target quantity in the reference sample (A1) based on the following equation: ΔΔC_T)test sample(_= [C_T (target)_ - C_T (VRN1)_] _(test sample)_ – [C_T (target)_ - C_T (VRN1)_] _(reference sample)_. Therefore, RQ (the relative copy number) = (2 × primer efficiency)^-ΔΔCT^ [29].

**Table 1:** Transposable element families analyzed in this study

Class^a^	Order^b^	Superfamily^c^	Family	Size (bp)
I	LTR	*Gypsy*	*Fatima*	9997
I	LTR	*Gypsy*	*Latidu*	13,068
I	LTR	*Unknown*	*Veju*	2530
I	Non-LTR	*SINE*	*Au*	181
II	TIR	*Mutator*	*Apollo*	866
II	TIR	*CACTA*	*Balduin*	11,744
II	TIR	*Tc1/Mariner*	*Eos*	354
II	TIR	*Tc1/Mariner*	*Fortuna*	353
II	TIR	*Tc1/Mariner*	*Tantalos*	257
II	TIR	*Tc1/Mariner*	*Minos*	236
II	TIR	*Tc1/Mariner*	*Aison*	219
II	TIR	*Tc1/Mariner*	*Oleus*	150

^a^I = class I- RNA elements, II = class II- DNA elements

^b^LTR- long terminal repeat retrotransposon, TIR- terminal inverted repeat

^c^Based on: TREP (http://botserv2.uzh.ch/kelldata/trep-db/index.html) and giri-repbase (http://www.girinst.org/repbase/update/browse.php). All the *Tc1/Mariners* are Inverted–Repeat Transposable Elements (MITEs)

For miniature elements (MITEs and *Au*) absolute copy numbers were calculated, using absolute numbers of elements retrieved from the bread wheat genome draft [30]. QPCR was performed to assess the RQ of these elements in bread wheat (accession TAA01, cultivar Chinese Spring) and in a wild emmer wheat reference sample, then the RQs were normalized to their ploidy levels, as the tetraploid accession used as reference sample has two copies of VRN1 gene, while the hexaploid bread wheat has three copies of VRN1. Absolute numbers of each element were calculated in the reference sample by multiplying the ratio of its relative quantity to that of *Triticum aestivum* by the copy number (CN) of *T. aestivum* retrieved from the bread wheat genome draft for each MITE using the MAK software [31]: (RQ_sample_/RQ_TAA01_) × Cnt_*. aestivum*_.

The resulting data was analyzed by using one-way analysis of variance (ANOVA); when a significant variation in average copy numbers of a TE family was detected by ANOVA, Tukey’s post hoc test was performed (*P* < 0.05 was set as the level of statistical significance). The statistical tests were performed using STATISTICA [32] software.

### Transposon display (TD)

TD is a modification of the AFLP technique, allowing a genome-wide analysis of transposon insertion sites [33, 34]. The TD reaction was performed according to a previously published protocol [33]. Briefly, genomic DNA was treated with *Mse*I restriction enzyme, and the restriction fragments were ligated to adaptors, which include an overhang complementary to the overhang produced by *Mse*I. A pre-selective PCR reaction was performed using one primer complementary to the adaptor sequence and a second primer designed from the transposon sequence. Selective amplification was carried out with the addition of three arbitrary nucleotides at the 3′ end of the adaptor primer and a ^32^P–labeled transposon primer. Here we have analyzed the insertional patterns of six Miniature Inverted–repeat Transposable Elements (MITEs) using a specific primer for each one of the MITE families (details on primers and adaptor sequences are presented in Additional file 1: Table S2). The selective PCR products were separated on denaturing 6% polyacrylamide gel. The gel was then removed on Whatman® paper, dried and exposed to X-ray film for 8 h, up to a week (depending on the β-emission intensity). Each TD band represents an insertion site that was clearly visible on the X-ray film. A matrix was built based on the presence (1) or absence (0) of a band in each accession for further analyses. The insertional polymorphism levels were calculated for each TE family in each population as the ratio between the number of polymorphic bands (bands present in some accessions and absent from other accessions of a population) and the total number of bands present in the population. Finally, TD bands were extracted from polyacrylamide gels, reamplified using the same PCR conditions as in the TD selective PCR reaction, and sequenced, as previously described [24].

### Computer-assisted analysis

Annotation and retrieving of TE sequences and their flanking sequences was performed using the genomic DNA, cDNA, coding sequence (CDS) and non-coding RNA (ncRNA) databases of different grass genomes taken from *EnsemblPlants* (http://plants.ensembl.org) and expressed sequence tag (EST) databases taken from *PlantGDB* (http://www.plantgdb.org/prj/ESTCluster/). The annotation was performed using BLAST+ standalone version 2.2.3 with an *E*-value of 10^−10^ [35].

### Phylogenetic analysis

Primer6 software [36], version 6.1.6 was used to construct hierarchical agglomerative clustering phylogenetic trees according the insertional polymorphism of each MITE family based on TD patterns, as described previously [24]. Primer6 software performed hierarchical agglomerative clustering analysis of each matrix with Bray-Curtis similarity and used the similarity profile (SIMPROF) test on each node to assess the statistical significance of the phylogenetic trees. SIMPROF calculates a mean profile by randomizing each variable’s values and re-calculating the profile. The *pi* statistic is calculated as the deviation of the actual resemblance profile of the resemblance matrix with the mean profile. This is compared with the deviation of further randomly-generated profiles to test for significance. In phylogenetic trees, black lines indicate statistically significant clusters (*p* < 0.05), and red lines indicate insignificant clusters.

### Site-specific PCR

Site-specific PCR primers (Additional file 1: Table S3) for examination of single TE insertion sites were designed using Primer3 software (http://bioinfo.ut.ee/primer3-0.4.0/primer3/). PCR reactions were prepared using 13.2 μl of Ultra Pure Water (HyLabs), 2 μl of 10× Taq DNA polymerase buffer C (EURx), 0.8 μl of 25 mM MgCl_2_ (EURx), 0.8 μl of 2.5 mM dNTPs, 0.2 μl of Taq DNA polymerase (5 U/μl, EURx), 1 μl of each site-specific primer (50 ng/μl) and 1 μl of genomic DNA (50 ng/μl) of each wild emmer wheat accession. The PCR conditions for these reactions were: 94°c for 3 min, repeat (94°c for 1 min, 57°c for 1 min, 72°c for 1 min) 30 times and 72°c for 3 min. 10 μl of PCR products were run on 1.5% agarose gels and visualized with ethidium bromide (Amresco). The expected product sizes were determined against a DNA size standard (100 bp ladder, SMOBiO).

### Gene expression analysis using real-time RT-PCR

Complementary DNA (cDNA) for gene expression analysis was prepared using 5× All-In-One RT MasterMix (ABM®), in 20 μl reaction volumes. Each reaction contained 4 μl of MasterMix and 2 μg of RNA in 16 μl of Ultra Pure Water (HyLabs). The reactions were incubated at 42 °C for 15 min. Each cDNA sample was tested using a site-specific PCR reaction with primers from two adjacent exons of the *Actin* gene, giving different amplification products for cDNA and genomic DNA and no DNA contamination was detected. The expression levels of five genes were tested by real-time RT-PCR. Primers specific to the cDNA sequence of each gene (Additional file 1: Table S4) were designed using Primer Express v2.0 software. For genes containing multiple exons, one of the primers was complementary to an exon-exon junction in order to make the reaction less sensitive to possible genomic DNA contamination of RNA samples. RT-PCR reactions were performed by a 7500 Fast Real-Time PCR system and analyzed using the 7500 version 2.0.5 software (Applied Biosystems). Each reaction contained: 7.5 μl KAPA SYBR FAST qPCR Master Mix, 0.3 μl ROX Low (KAPA BIOSYSTEMS), 1 μl forward and 1 μl reverse primers (10 μM), 0.2 μl ddH_2_O and 5 μl template cDNA. The PCR conditions for these reactions were: 95°c for 3 min, repeat (95°c for 1 min, 60°c for 1 min) 40 times; in order to validate product specificity, a melting curve was performed and demonstrated a single specific product for each primer pair (see an example in Additional file 2: Figure S9).

Primer efficiency was tested by performing RT-PCR reactions with each primer pair on serial dilutions of template cDNA mix and producing a standard curve. Efficiency = [(10^–1/y^) – 1] × 100%, where y is the standard curve slope. Based on the standard curve, we chose the 50-fold dilution of cDNA to be used as template in RT-PCR reactions for gene expression analysis.

RT-PCR data was analyzed as described above using comparative 2^-ΔΔCT^ method, using *Actin* expression levels as endogenous control and one arbitrarily chosen accession as reference sample.

## Results

### Copy number variation of selected DNA and RNA elements in wild emmer wheat populations

**Fig. 1:**
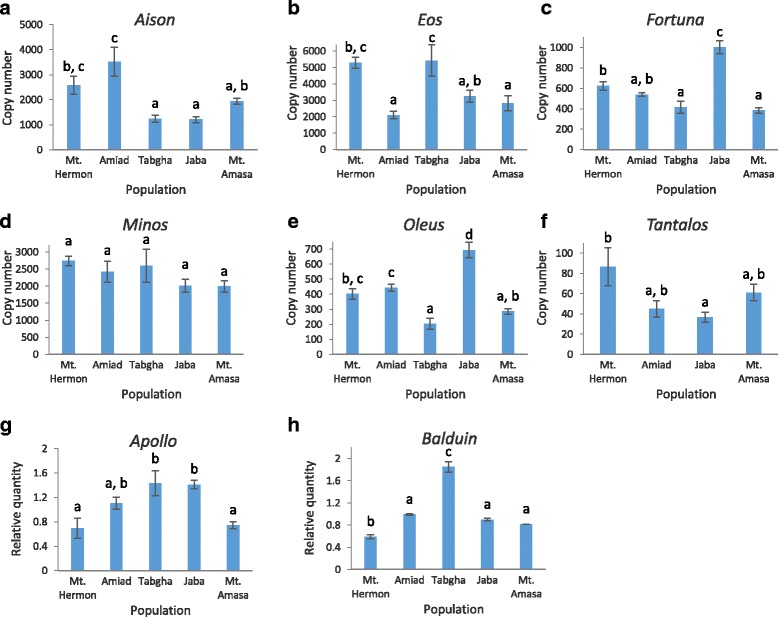
Quantification of DNA elements in wild emmer wheat populations (Mt. Hermon, Amiad, Tabgha, Jaba and Mt. Amasa), shown as the average of 9–10 accessions from each population. The analysis included six MITE families: **a**) *Aison*; **b**) *Eos*; **c**) *Fortuna*; **d**) *Minos*; **e**) Oleus; **f**) *Tantalos*; and two long DNA elements: **g**) *Apollo*; **h**) *Balduin*. Error bars represent standard errors. Letters above error bars visualize the results of Tukey test performed on the copy number data: populations with no significant differences between their mean copy numbers are listed in the same homogenous group sharing the same letter; the differences in mean copy numbers between populations not belonging to the same group are significant (*p* < 0.05)

**Fig. 2:**
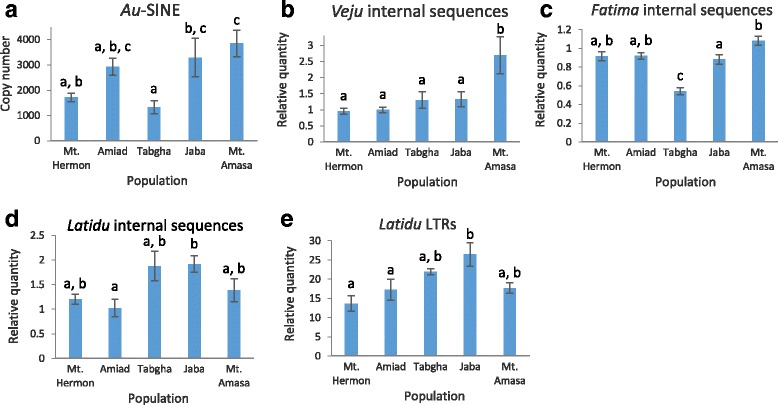
Quantification of retrotransposons in wild emmer wheat populations (Mt. Hermon, Amiad, Tabgha, Jaba and Mt. Amasa), shown as the average of 5–10 accessions from each population. **a**) Miniature retrotransposon *Au*. LTR-retrotransposons: **b**) *Veju*, **c**) *Fatima* (internal sequence), **d**) *Latidu* (internal sequence), **e**) *Latidu* (LTR). Error bars represent standard errors. Letters above error bars visualize the results of Tukey test performed on the copy number data: populations with no significant differences between their mean copy numbers are listed in the same homogenous group sharing the same letter; the differences in mean copy numbers between populations not belonging to the same group are significant (*p* < 0.05)

Because over 80% of the wheat genome is made up by TEs, they might have a prominent impact on the genetic diversity in wild emmer wheat populations. Thus, in this study, we have evaluated the copy numbers of several TE families representing different classes and different superfamilies, in each one of the 50 accessions. To this end, the copy numbers of four class I families (*Fatima*, *Latidu*, *Veju* and *Au*) and eight class II (*Apollo*, *Balduin*, *Eos*, *Fortuna*, *Tantalos*, *Minos*, *Aison* and *Oleus*) families (Table 1) were measured in all accessions. We used real-time quantitative PCR to assess the copy number of each one of the 12 TE families in each one of the 50 accessions, using specific primers for each TE family (Additional file 1: Table S1). The quantity of each TE family amplified by qPCR was normalized to *VRN1*, a gene with a single copy in each wheat genome [26–28], and then to one accession (A1 accession from Amiad population, see Additional file 2: Figures S1 and S2) that was arbitrarily chosen, and set as 1 in order to allow relative comparison among all accessions. For miniature TE families (MITEs and *Au*), absolute numbers were estimated using normalization to the copy number of each family in *T. aestivum* genome draft [30]. The copy numbers of long elements in wild emmer wheat accessions are presented here as relative quantities based on the qPCR analysis. Finally, for each one of the analyzed TE families, the average copy numbers in each one of the five populations were calculated separately (Figs. 1 and 2). The statistical significance of variation of average copy numbers among populations was analyzed by using one-way analysis of variance (ANOVA), followed by Tukey’s post hoc test (*p* < 0.05 was considered statistically significant).

Copy numbers in each accession are shown in Additional file 2: Figure S1 (for DNA elements), and Additional file 2: Figure S2 (for retrotransposons). Note that standard deviation (SD) in all realtime qPCRs were calculated based on three replicates from each one of the accessions.

#### Copy number variation of DNA elements (class II)

Except for *Minos*, the five MITE families (*Eos*, *Fortuna*, *Tantalos*, *Aison* and *Oleus)* showed statistically significant (p < 0.05) copy number variation among accessions of the 5 populations of emmer wheat (Fig. 1): (1) *Aison* showed the highest average copy number of 3528 insertions in accessions collected from Amiad population, significantly higher than in other populations (Fig. 1a), while accessions of Tabgha and Jaba populations showed the smallest average copy numbers of *Aison*, with 1224 and 1210 copies, respectively. In addition, Mt. Hermon and Mt. Amasa accessions showed intermediate average copy number of *Aison*. Furthermore, significant variation among accessions within each population was observed (Additional file 2: Figure S1a), ranging from close to 1.5-fold differences between some accessions within Mt. Amasa and Mt. Hermon populations and over 4.5-fold differences within Amiad population. (2) *Eos* showed the lowest average copy number of 2104 in accessions of the Amiad population (Fig. 1b), significantly lower than in accessions of other populations. The highest average copy numbers were observed in Mt. Hermon and Tabgha populations (5305 and 5417, respectively), and while the average copy numbers in these two populations were similar, the standard error demonstrates high internal variation in Tabgha accessions compared to Mt. Hermon. Jaba and Mt. Amasa accessions showed intermediate average copy numbers. The copy number differences within most populations ranged between 2-fold to 4-fold (Additional file 2: Figure S1b). (3) *Fortuna* showed the lowest average copy number of 384 in accessions of Mt. Amasa, while the highest average copy number of 1003 was observed in Jaba (Fig. 1c). The average copy numbers in accessions of Tabgha were close to Mt. Amasa, in Mt. Hermon and Amiad higher average copy numbers were observed. In addition, no significant copy number variation was observed within accession of the Amiad population, while in other populations the greatest differences between accessions ranged between 2-fold (Mt. Hermon) and 6-fold (Tabgha) (Additional file 2: Figure S1c). (4) *Minos* average copy numbers were found to range between 2742 in Mt. Hermon and 1989 in Mt. Amasa (Fig. 1d), but no significant variation between populations was seen. The copy numbers of *Minos* were not uniform within populations, the highest copy number differences within populations ranged between 1.5-fold (in the Mt. Hermon population) and 2-fold (in the Tabgha population) (Additional file 2: Figure S1d). (5) The average copy numbers of *Oleus* ranged between 202 in accessions of Tabgha and 694 in accessions of Jaba. *Oleus* copy numbers in accessions of Mt. Amasa were close to those in Tabgha, while Mt. Hermon and Amiad show intermediate copy numbers of this element (Fig. 1e). Significant copy number variations between accessions in each population were observed (Additional file 2: Figure S1e), with over 6-fold differences between accessions of the Tabgha population. (6) *Tantalos* copy numbers, tested in 4 populations, were relatively low, ranging between 37 copies in Jaba and 87 in Mt. Hermon (Fig. 1f). Accessions of Amiad and Mt. Amasa showed intermediate copy numbers of 45 and 61, respectively. Significant variation within populations was observed, with the lowest being a 4-fold difference between Mt. Amasa accessions and the highest a 10-fold difference between Amiad accessions (Additional file 2: Figure S1f). The average copy numbers of two long DNA elements (*Apollo* and *Balduin*, see Table 1) were also found to be statistically significant among the 5 populations (Fig. 1g, h, respectively). For *Apollo*, the highest relative quantity was observed in accessions from Tabgha and Jaba populations, while the lowest relative quantity (~2-fold less) was observed in Mt. Hermon and Mt. Amasa populations. Furthermore, the observed relative quantity in each accession shows that the variation among accessions reaches up to 6-fold differences (Additional file 2: Figure S1 h). The highest average relative quantity of *Balduin* was observed in accessions of Tabgha ~3-fold higher than in Mt. Hermon and 2-fold higher than in Amiad (Fig. 1h). In addition, the average relative quantity in Jaba and Mt. Amasa were close to those in Amiad. Furthermore, no significant variation was seen among accessions within each population indicating low level of *Balduin* activity in emmer wheat.

#### Copy number variation of RNA elements (class I)

Here we have measured the copy number or relative quantity of 4 retrotransposons (*Au*, *Veju*, *Fatima* and *Latidu*) representing different superfamilies (Table 1). As we have mentioned above, we were able to estimate the absolute copy number of *Au* because of its short sequence (181 bp, Table 1) which can be retrieved from the genome draft accurately. The average copy numbers of *Au* were significantly different among populations, ranged between 1321 in Tabgha and to 3853 in Mt. Amasa (Fig. 2a). In addition, a significant variation in copy number among accessions within each one of the five populations was observed (Additional file 2: Figure S2a), indicating high dynamics of *Au* in wild emmer wheat. The relative quantities of three LTR retrotransposons (*Veju*, *Fatima* and *Latidu*) were measured by real-time qPCR using primers specific to the internal sequence that allow the amplification of intact and truncated elements in the genome. In addition, for further validation of the relative quantity, *Latidu* was quantified using primers designed specifically from the LTR sequence, in order to also estimate instances of solo-LTRs. Note that the relative quantities of both *Latidu* internal sequences and *Latidu* LTRs were normalized to the same reference sample in order to allow comparison between them. To this end, the relative quantity of *Veju*, a non-autonomous LTR retrotransposon, was observed in the 50 accessions of the 5 wild emmer wheat populations, and was found significantly different among accessions (Additional file 2: Figure S2b). The average relative quantity in each population was compared and displayed significant differences (Fig. 2b), while the highest average quantity was observed in Mt. Amasa, ~2-fold higher than the average quantity in the Mt. Hermon and Amiad populations. *Fatima* is a long (approximately 10 kbp, Table 1) LTR retrotransposon, previously reported to be very abundant in the wheat genome [37]. The relative quantity of *Fatima* was measured in all 50 accessions and significant differences were found (Additional file 2: Figure S2c). Finally*,* the relative quantity of *Latidu* was measured in 26 accessions from 5 populations and significant differences were found (Additional file 2: Figure S2d). In addition, the average relative quantities of *Latidu* vary significantly between populations, while the highest quantities were observed in Tabgha and Jaba populations, and the lowest were observed in Amiad and Mt. Hermon (Fig. 2d). Note that similar patterns were obtained when the quantity of *Latidu* LTRs was tested (Fig. 2e, and Additional file 2: Figure S2e).

### Insertional patterns of MITEs in wild emmer wheat accessions

**Fig. 3:**
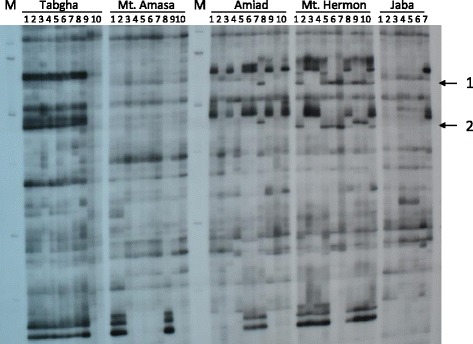
TD gel visualizing insertional patterns of *Aison* (MITE) in 7–10 accessions of 5 wild emmer wheat populations using the *Aison*-specific primer and M-CTA (see Additional file 1: Table S2). Each lane represents one accession and each band represents a single insertion site. M denotes the size marker (100 bp). Examples of population-specific/unique bands are indicated by arrows and numbers: 1 – population-specific insertion (exists in Amiad and Mt. Hermon populations but not in others), 2 – population-unique insertion (exists only in Mt. Hermon)

The significant variation in copy numbers of most examined TEs observed in wild emmer wheat accessions hinted that proliferation of TEs might occur in a population-specific manner. Thus, we have analyzed the insertional patterns in all accessions of the 5 populations using the Transposon Display (TD) technique. The TD analysis allowed detection of population- specific/unique transposon insertional patterns. In this analysis, we have focused on MITEs because they were previously reported to show high dynamics in wheat [27, 28, 38]. We have analyzed the insertional patterns of the six MITE families (Table 1) in 7–10 accessions of each population, using TD. Each lane in the TD pattern presents a subset of MITE/flanking-containing sequences (Fig. 3), indicating specific insertion sites of a specific MITE family in one accession. For each MITE family, 70–115 insertions (overall 557 MITE insertions) were analyzed by TD (Table 2). Insertional polymorphism levels in each wild emmer wheat population were calculated for each one of the six MITE families based on the number of polymorphic insertions among accessions of each population (Table 2). Note that an insertion was considered polymorphic if it is absent in at least one accession. Of the 557 total MITE insertions, 400 were polymorphic, namely 78.8% of the insertions were polymorphic in all tested accessions of wild emmer wheat. This high level of polymorphism might indicate that MITEs might have retained their activity in natural populations of emmer wheat. The overall insertional polymorphism levels of MITEs ranged between 38.6% (*Minos*) and 91.9% (*Aison*). The polymorphism levels of MITEs vary among populations, for example *Aison* polymorphism levels are between 79.5% in Amiad and 41.7% in Mt. Amasa, possibly indicating different activity levels in the different populations.

**Table 2:** Insertional polymorphism levels of 6 MITEs in wild emmer wheat populations as observed in TD

TE^a^ family		Population^b^					Total^c^
		Mt.Hermon	Amiad	Tabgha	Jaba	Mt.Amasa	
*Aison*	Polymorphic bands	50	62	57	45	25	92
	Total number of bands	72	78	72	70	60	99
	Polymorphism level (%)	69.4	79.5	79.2	64.3	41.7	91.9
*Eos*	Polymorphic bands	37	39	50	26	23	69
	Total number of bands	75	75	79	66	68	92
	Polymorphism level (%)	49.3	52.0	63.3	39.4	33.8	75.0
*Fortuna*	Polymorphic bands	52	51	58	54	41	61
	Total number of bands	65	65	68	68	54	70
	Polymorphism level (%)	80.0	78.5	85.3	79.4	75.9	87.1
*Minos*	Polymorphic bands	18	23	13	15	9	34
	Total number of bands	81	83	76	80	79	88
	Polymorphism level (%)	22.2	27.7	17.1	18.8	11.4	38.6
*Oleus*	Polymorphic bands	36	28	27	38	20	48
	Total number of bands	81	80	79	85	76	92
	Polymorphism level (%)	44.4	35.0	34.2	44.7	26.3	52.2
*Tantalos*	Polymorphic bands	56	48	63	70	43	96
	Total number of bands	100	95	99	107	92	116
	Polymorphism level (%)	56.0	50.5	85.9	65.4	46.7	82.8

^a^TD reactions were performed for 6 MITE families using: (1) two primer combinations, *Aison*-specific primer/M-CTA and *Aison*-specific primer/ M-CTC; (2) two primer combinations*, Eos*-specific primer/M-CTA and *Eos*-specific primer/M-CTG; (3) *Fortuna*-specific primer/M-CTA; (4) *Minos*-specific primer/M-CTA and *Minos*-specific primer/M-CTG; (5) *Oleus*-specific primer/M-CTA and *Oleus*-specific primer/M-CTG; (6) *Tantalos*-specific primer/M-CTA and *Tantalos*-specific primer/M-CTG. See Additional file 1: Table S2 for details on primer sequences

^b^TD band was considered polymorphic when it was present/or absent in at least one accession. Total number of bands is the sum of polymorphic and monomorphic bands

^c^Based on the analysis of all 50 accessions together, indicating the overall level of polymorphism in the collection set

**Fig. 4:**
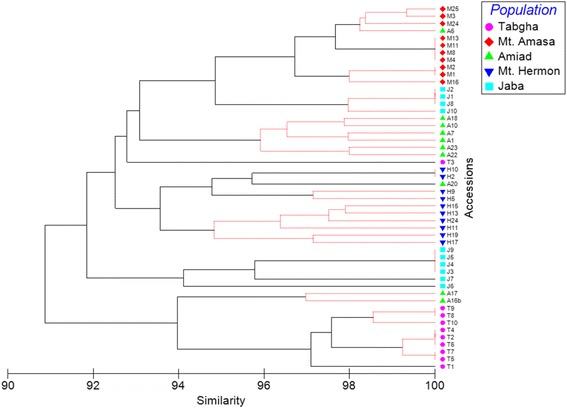
Phylogenetic tree generated by hierarchical agglomerative clustering based on 88 TD bands of *Minos* in 50 accessions from 5 wild emmer wheat populations: Mt. Hermon, Amiad, Tabgha, Jaba and Mt. Amasa. The index (top right) shows the collection site of each accession. The black lines indicate significant separation, while red lines indicate insignificant separation. The level of similarity is indicated on the bottom

Phylogenetic trees generated by hierarchical agglomerative clustering were built based on the insertional patterns of the six MITE families and showed that accessions were significantly (*p* < 0.05) clustered based on their geographical location (Fig. 4 and Additional file 2: Figures S3-S7), indicating population-specific MITE proliferation in wild emmer wheat. The phylogenetic tree based on *Minos* insertional patterns (Fig. 3) showed that all accessions of Mt. Amasa and Mt. Hermon and most accessions of Tabgha significantly clustered according to their geographical location. The phylogenetic trees built based on the data of the rest of the tested MITE families (*Eos*, *Aison*, *Fortuna*, *Oleus* and *Tantalos*, Additional file 2: Figures S3-S7) demonstrated different degrees of significant population-specific clustering and intra-population separation, possibly indicating different degrees of activity.

### Polymorphic MITE insertions into genes cause allelic variation in a population-specific/unique manner

Interestingly, 181 of the polymorphic insertions (17.9% of the analyzed 557 MITE insertions) were considered population-specific insertions because they were absent in all accessions of at least one or more populations and present in at least one accession of each one of the other populations (for example see band #1 in Fig. 3). In addition, 38 (6.8%) insertions were found to be population-unique, namely, the insertion is present in one population only and absent in all accessions of the other populations (for example see band #2 in Fig. 3).

In order to characterize in detail population-specific/unique insertions, we have extracted 52 bands from polyacrylamide gels, reamplified them using the same conditions of the selective PCR reaction used in TD assay, and then sequenced them. The sequence of the 52 TD bands showed that all include MITE/flanking sequences. Annotations of the flanking sequences of the 52 revealed that most sequences (36 of the 52) hit noncoding sequences, while 6 hit known TEs, and the remaining 10 hit predicted protein-coding sequences (Table 3). This data showed that population-specific/unique TE insertions might be located within wheat genes and might affect the structure (allelic variation) or the expression of the gene in different populations.

**Table 3:** Detailed analysis of genes harboring polymorphic insertions of MITEs

Gene^a^	Gene product^b^	TE family^c^	TE insertion location^d^	Insertions in population^e^					Gel image^f^
				H	A	T	J	M	
TRIUR3_24471	a sterol-dependent membrane protein, At3g47200-like	*Aison*	400 bp upstream, genome B	2	0	0	0	0	Fig. 6a
TRIUR3_29094	integral membrane component	*Aison*	Intron 4, genome A	0	1	2	0	0	Fig. S8a
Traes_2BS_2453C5E6B	Response to oxidative stress; peroxidase activity	*Aison*	Intron 3, genome B	2	1	2	2	2	Fig. S8b
Traes_7BL_2E24532BD	RNA binding, ribosomal protein	*Aison*	Intron, genome B	2	1	1	1	2	Fig. S8c
FJ640556.1	AIP2.2 - ABI3 (abscisic acid-insensitive 3)-interacting protein	*Au*	Intron 3, genome B	2	1	1	1	2	Fig. S8d
Traes_1BL_DD7D021A7	Ubiquitin-protein ligase	*Au*	Intron 5, genome B	1	1	0	0	0	Fig. S8e
TRIUR3_22200	ER membrane protein, transferring acyl groups other than amino-acyl groups, metabolic process	*Eos*	Intron 4, genome A	2	1	2	1	1	Fig. S8f
		*Thalos*	Intron 1, genome A	2	2	1	1	2	Fig. 6d
Traes_4BS_AAE1439D4	Nuclear-localised, ATP binding, transcription regulation	*Eos*	Exon (UTR), genome A	2	1	0	1	2	Fig. 6c
GU817319.1- locus tag - 2383A24.5	Predicted, homolog of Os01g0121600, integral membrane component	*Hades*	960 bp upstream, genome B	0	1	0	0	0	Fig. S8 g
Traes_3AS_94E185821	Calcium ion binding protein	*Minos*	2 bp downstream, genome A	2	1	1	2	2	Fig. S8 h
Traes_3AS_2755E639C	Uncharacterized protein	*Minos*	22 bp upstream, both genomes	1	1	1	0	0	Fig. S8i
Traes_7AL_0D3EF0026	Uncharacterized protein	*Minos*	Exon 2 (UTR), genome A	0	1	1	0	0	Fig. 6b
Traes_3B_5DEF2D3F1	Basic helix-loop-helix transcription factor	*Tantalos*	Intron 3, genome B	0	1	1	0	0	Fig. S8j

^a^Gene ID in EnsemblPlants database, or GenBank accession in NCBI

^b^Protein product function, based on annotation found in NCBI (for known genes), or prediction from EmsemblPlants

^c^MITE associated with genes

^d^Position of the given TE insertion in relation to gene features (the number of intron or exon for insertions within genes, or the distance in base pairs from the first or the last exon for insertions upstream of downstream to genes), and the genome (A, B or both) in which the given insertion was detected

^e^The presence/absence of TE insertion within/close to a gene in a given population: 0 – empty site in all accessions, 1 – full site in some accessions, 2 – full site in all accessions

^f^Figure or supplemental figure showing ssPCR gel image

**Fig. 5:**
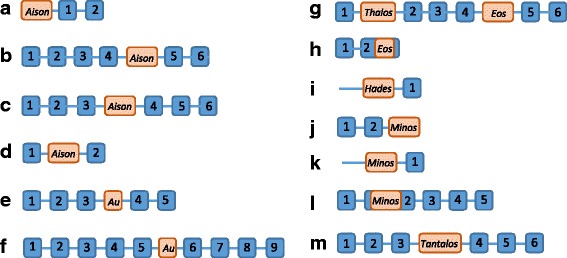
Schematic representation of the structure of 13 genes associated with 14 polymorphic TEs insertions. Blue boxes with numbers noted on them indicate exons, brown boxes with a TE family noted on them indicate TE insertions. **a**) TRIUR3_24471, **b**) TRIUR3_29094, **c**) Traes_2BS_2453C5E6B, **d**) Traes_7BL_2E24532BD, **e**) FJ640556.1, **f**) Traes_1BL_DD7D021A7, **g**) TRIUR3_22200, **h**) Traes_4BS_AAE1439D4, **i**) GU817319.1, **j**) Traes_3AS_94E185821, **k**) Traes_3AS_2755E639C, **l**) Traes_7AL_0D3EF0026, **m**) Traes_3B_5DEF2D3F1

**Fig. 6:**
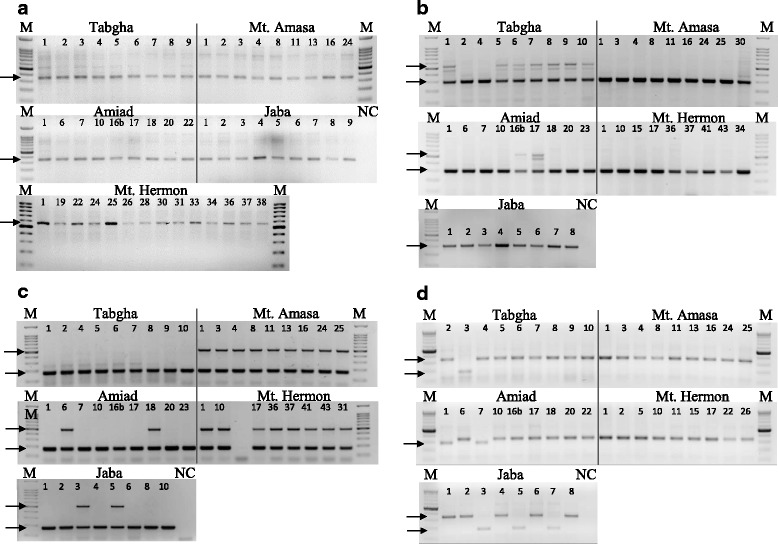
Examples of site-specific PCR analyses with TE-flanking primers (**a**, **d** – genome-specific primers; **b**, **c** – primers complementary to both A and B genomes) in 5 populations of wild emmer wheat, showing polymorphic TE insertions. Full and empty sites are indicated by arrows. M denotes size marker. **a**) Population-unique insertion of *Aison* 400 bp upstream to TRIUR3_24471 gene, a 564-bp band indicating that a full site is present in all accessions of Mt. Hermon (bottom), 346 bp band indicating that an empty site is present in all accessions of Tabgha and Mt. Amasa populations (top) and of Amiad and Jaba populations (middle). **b**) Insertion of *Minos* in exon 2 of the Traes_7AL_0D3EF0026 gene in some accessions of Tabgha (top right) and Amiad (middle right), where the upper band (537 bp) indicates a full site. The lower band (301 bp) is present in all accessions. **c**) Insertion of *Eos* in an exon of the Traes_4BS_AAE1439D4 gene, present in all examined accessions of Mt. Amasa (top right) and Mt. Hermon (middle right) populations and some accessions of Amiad (middle left) and Jaba (bottom), but not in Tabgha (top left). The upper band (500 bp) indicates a full site, the lower band present in all accessions (170 bp) indicates an empty site. **d**) Insertion of *Thalos* in intron 1 of the TRIUR3_22200 gene present in all accessions Mt. Amasa (top right), Amiad and Mt. Hermon (middle), and absent from one accession of Tabgha (top left) and some accessions of Jaba (bottom). The upper band indicates a full site (347 bp – intact TE, or 295 bp – truncated TE in 2 accessions of Amiad validated by sequencing). The lower band (185 bp) indicates an empty site

In order to focus on polymorphic MITE insertions within genes we have arbitrarily retrieved 60 MITE elements and their flanking sequences (100 bp from each side of the element) from the publicly available sequences of emmer wheat, which were inserted into known protein-coding genes, including those that were detected in TD analysis. We then performed site specific PCR to test whether an accession included a specific MITE insertion (full site) or not (empty site), using specific primers from MITE-flanking sequences. We observed 14 cases of polymorphic insertions into introns (8 cases), exons (2 cases, upstream or downstream to predicted coding sequence - UTR regions) or adjacent (4 cases, up to 1 kb upstream/downstream) to genes among emmer wheat accessions (Table 3 and Fig. 5), while 8 cases were population-unique insertions (e.g., an insertion of an *Aison* element 400 bp upstream of a predicted gene encoding a *sterol-dependent membrane protein*, in all accessions collected from Mt. Hermon only, Fig. 6a), or population-specific insertions (e.g.: an insertion of a *Minos* element in exon 2 of an uncharacterized protein in accessions from Amiad and Tabgha only, Fig. 6b; an insertion of *Eos* in an exon of a *nuclear-localized, ATP binding, transcription regulation* gene in all accessions of Mt. Amasa and Mt. Hermon populations, and some accessions of Amiad and Jaba, but not in Tabgha, Fig. 6c; and an insertion of *Thalos* in intron 1 of the ER membrane protein, transferring acyl gene present in all accessions of Mt. Amasa, Amiad and Mt. Hermon, and absent from one accession of Tabgha and some accessions of Jaba, Fig. 6d). Detailed analysis of the 14 polymorphic insertions, including the exact location of the TE insertion and the insertional patterns in wild emmer wheat accessions is presented in Table 3. In addition, site-specific PCR analysis is also shown in Additional file 2: Figure S8. Note that two polymorphic insertions of *Thalos* and *Eos* were found in introns of the same gene (Table 3, Fig. 5g). Our data indicate that insertions of MITEs into or adjacent to protein-coding genes cause population-specific allelic variation in wild emmer wheat. In 13 of the 14 cases, the polymorphic MITE insertions were present only in one of the two subgenomes (6 insertions in AA subgenome and 7 insertions in BB subgenomes) of emmer wheat, while only one polymorphic insertion was present in both subgenomes (Table 3).

### Expression analysis of genes that harbor polymorphic MITE insertions

**Fig. 7:**
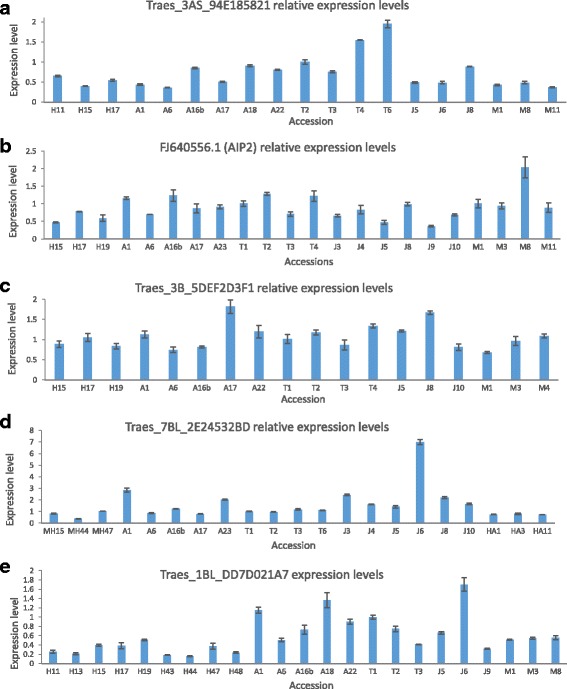
Relative expression levels of 5 genes in young leaves of wild emmer wheat from 5 populations (H – Mt. Hermon, A – Amiad, T – Tabgha, J – Jaba, M – Mt. Amasa). Error bars represent standard deviations of 3 replicates in RT-PCR reactions. **a**) Traes_3AS_94E185821; **b**) FJ640556.1 (AIP2); **c**) Traes_3B_5DEF2D3F1; **d**) Traes_7BL_2E24532B; **e**) Traes_1BL_DD7D021A7. Expression levels of each gene in each accession are shown relative to accession T2 in panel a, and accession T1 in panels b-e

The expression of genes might be associated with TE insertions [22, 39]. As such, the observed allelic variation in protein-coding genes caused by TE insertion in wild emmer wheat might impact the expression of those genes. Note that most of the genes that were found to harbor polymorphic MITE insertions were not studied before in wheat. In addition, we found that the genes are highly conserved (over 99% similarity at the predicted exons sequences) between the two subgenomes (AA and BB) of emmer wheat. Thus, because the polymorphic MITE insertions were found in only one of the subgenomes in most cases (13 out of 14; Table 3), it was challenging to test the expression of those genes in a genome-specific manner. To this end, we have successfully examined the expression of five genes (Ensembl or gene bank accessions: Traes_7BL_2E24532BD, FJ640556.1, Traes_1BL_DD7D021A7, Traes_3AS_94E185821, Traes_3B_5DEF2D3F, Table 3) in young leaves of all tested wild emmer wheat accessions from the five populations. Real-time RT-PCR was performed using cDNA prepared from RNA extracted from young leaves of wild emmer wheat accessions. Primers were designed from exon-exon junctions to ensure that there are no artifacts from genomic DNA amplifications, and in all cases, the primer efficiency was 100% (Additional file 1: Table S4, Additional file 2: Figure S9). Thus, the relative expression levels of the five genes were tested in 3–9 accessions from each one of the five wild emmer wheat populations. Note that in order to measure standard errors, the RT-PCR experiments were done in triplicates, and the expression levels (relative to the normalized gene, *Actin*) of all accessions were presented in the graph by comparing their expression levels to a reference (accessions T1 or T2), whose expression level was set as 1. Interestingly, all five genes showed significant differences in their expression levels among the different accessions of emmer wheat (Fig. 7). The expression levels of Traes_3AS_94E185821, a gene that encodes a predicted calcium-ion binding protein ranged between 0.3 fold (the expression of T2 accession was set as 1) in some accessions up to 2 fold in others (Fig. 7a), where the lower expression levels were observed in the accessions from Mt. Hermon and Mt. Amasa. The expression levels of FJ640556.1, a gene encoding for *AIP2.2 - ABI3 (abscisic acid-insensitive 3*)-interacting protein, ranged between 0.3 and 2 fold (the expression of T1 accession was set as 1) in the various accessions (Fig. 7b). The expression levels of Traes_3B_5DEF2D3F1, a gene encoding for a predicted *basic helix-loop-helix* transcription factor ranged between 0.5 and 1.8 fold (the expression of T1 accession was set as 1) in the various accessions (Fig. 7c). The expression levels of Traes_7BL_2E24532BD, a gene encoding for a predicted *RNA binding, ribosomal protein* ranged between 0.8 and 7.5 fold (the expression of T1 accession was set as 1) in the various accessions (Fig. 7d). Finally, the expression levels of Traes_1BL_DD7D021A7, a gene encoding for a predicted *Ubiquitin-protein ligase* ranged between 0.2 and 1.7 fold (the expression of T1 accession was set as 1) in the various accessions (Fig. 7e).

## Discussion

One of the consequences of wheat domestication and breeding programs is the loss of genetic diversity in commercial wheat cultivars [3, 40–42]. There are huge efforts in recent decades to restore genetic diversity to modern wheat cultivars from wild emmer wheat, the wild progenitor of all domesticated wheat species (the mother of wheat). The potential of wild emmer as a source of genes for biotic and abiotic stress tolerance has been widely recognized [43–45]. While many studies have been carried out on the phenotypic and genetic variations in natural populations of wild emmer wheat, almost nothing is known about the genome dynamics of wild emmer wheat in different climate conditions.

In this study, we have performed detailed analyses of TE dynamics in natural habitats of wild emmer wheat accessions, including: copy number variation, insertional patterns, allelic variation in protein-coding genes caused by TEs, and the impact of population-unique/specific TE insertions on gene expression. We found significant variation of the copy numbers of 12 TE families representing both TE classes (DNA and RNA elements). In addition, we found population-unique insertional patterns of TEs based on the phylogenetic analysis of six MITE families in the 50 accessions collected from five isolated geographical sites. About 18% of the analyzed 557 MITE insertions were considered population-specific and about 6% of the MITE insertions were population unique. Some of those population-specific/unique insertions were inserted into introns or exons of protein-coding genes and caused allelic variation in the wild emmer wheat. In accordance, we have validated the expression of some protein-coding genes that harbor polymorphic MITE insertions in wild emmer wheat accessions and differential expression was seen.

### Genetic polymorphism and TE dynamics

In order to evaluate the contribution of TEs to the genetic variation in natural populations of wild emmer wheat we estimated the overall level of genetic polymorphism in wild emmer populations using TD [33, 46], an unbiased and a reliable technique (Fig. 3). It is important to note that although in some cases the common garden conditions would be different from the natural conditions for some populations, but the conditions used in the greenhouse are not stressful to any of the populations, therefore they should not affect the veracity of the conclusions.

Overall, 557 MITE insertions of 6 MITE families were analyzed in this study in all accessions of the five populations. The insertional polymorphisms varied dramatically between the MITE families, where some MITE families showed a relatively low polymorphism level (such as *Minos*, average polymorphism level of 38.6%, Table 2), and other families showed very high polymorphism level (such as *Aison*, average polymorphism level of 91.9%, Table 2). The TD data indicate that TE dynamics might largely contribute to the genetic biodiversity in emmer wheat because TEs comprise over 80% of the wheat genome and therefore might be responsible for most genetic variation between accessions.

Phylogenetic trees based on TD data significantly (*p* < 0.05) clustered the wild emmer wheat accessions based on geographical location (Fig. 4 and Additional file 2: Figures S3-S7).

The TD data support previous findings of population-specific genetic clustering [4, 12]. The significant separation between accessions within populations also correspond with previous reports regarding micro-scale adaptive genetic differentiation [14, 47], and limited gene flow between predominantly self-pollinating plants [48] is likely to preserve existing genetic patterns.

Do the significant population-unique insertional patterns observed here indicate MITE activity in natural populations of wild emmer wheat? Assuming that each population starts from a certain number of individuals with a certain set of genotypes changing under selection and other evolutionary constrains, and provided all examined MITEs existed before the populations were established, a certain number and pattern of each MITE was linked to each of the initial genotypes. Therefore, TE insertional polymorphism alone might not be sufficient evidence of transposon activity in natural populations, because this may be explained by founder effect. In addition, it is difficult to point to the exact reasons for differential MITE activity, because they are non-autonomous elements requiring suitable active autonomous DNA elements in order to be mobilized, and those are still unknown in wheat. It was previously reported that TEs can be mobilized by stress conditions, and such conditions could occur simultaneously or at different times in different habitats.

Differential proliferation of TEs in wild emmer wheat populations might lead to huge copy number variation among accessions. Here we observed significant copy number variation of 12 TE families, including DNA and RNA elements (Table 1), in the 50 accessions collected from the five isolated geographical sites (Fig. 1 and Fig. 3). The question, whether those significant differences in TE copy number might affect genome size, was considered and tested by measuring the genome size of wild emmer wheat accessions collected from four populations, using the Fluorescence-Activated Cell Sorting (FACS) technique (see details in Additional file 2: Figure S10). The FACS data revealed a similar genome size for accessions collected from different populations, indicating that the variation in TE copy number did not massively affect the genome size of wild emmer wheat. One explanation is that the copy number variation observed here was in all cases TE family-specific, meaning that, for example, while the copy number of one TE family was relatively high in a specific population, another TE family showed a relatively low copy number in the same population.

### Allelic variation of genes harboring MITEs

The TD data motivated us to search for population-specific/unique insertions in protein-coding genes and we observed many of those cases (Figs. 6 and 7, and Additional file 2: Figure S8). Out of the 14 examined polymorphic insertions: 8 were found in introns, 3 upstream to genes in distances between 22 bp to 960 bp, one insertion was 2 bp downstream to a gene, and two insertions occurred within exons but upstream or downstream to predicted coding sequences (Table 3). These insertions can possibly be neutral or can alter gene function in different ways depending on their locations: TEs within introns can possibly interfere with the splicing process, insertions upstream or downstream to a gene can affect regulatory sequences, and insertions into UTR regions do not change the coding sequence but can possibly affect transcript stability. In order to examine whether each particular insertion alters expression levels, genome-specific expression analysis will be needed, because the insertional polymorphism exists in one of the two genomes, and the expression can occur from one or both genomes. Here we showed that protein-coding genes harboring polymorphic MITE insertions in wild emmer wheat had different expression levels among wild emmer wheat accessions (Fig. 7). It is still not fully clear whether the population-unique/specific polymorphic TE insertion on those genes impacts their expression and whether it is correlated with any adaptive value.

## Conclusions

We show here novel data on massive TE dynamics in natural populations of a very important organism — wild emmer wheat (the mother of all wheats). We showed that TE dynamics might affect genome structure and expression in a population-specific/unique manner. This data sheds lights on the role of TEs in shaping the genome of natural plant populations in different climate conditions, especially because TEs are the single largest component of most land plant genomes [49].

## Additional files

Additional file 1: Table S1. Primer sequences used for copy number variation analysis of TEs by real-time qPCR. **Table** **S2**. Adaptor and primer sequences used in Transposon Display (TD). **Table S3.** Primer sequences for site-specific PCR. **Table S4.** Primer sequences for site-specific PCR. (DOCX 28 kb)

Additional file 2: Figure S1. Quantification of DNA elements in accessions of 5 wild emmer wheat population. **Figure S2**. Relative quantities of long TEs in 5 wild emmer wheat populations. **Figure S3.** Phylogenetic tree generated by hierarchical agglomerative clustering based on 99 TD bands of *Aison.* **Figure S4.** Phylogenetic tree generated by hierarchical agglomerative clustering on 70 TD bands of *Fortuna.* **Figure S5.** Phylogenetic tree generated by hierarchical agglomerative clustering based on 92 TD bands of *Oleus.* **Figure S6.** Phylogenetic tree generated by hierarchical agglomerative clustering based on 116 TD bands of *Tantalos.* **Figure S7.** Phylogenetic tree generated by hierarchical agglomerative clustering based on 92 TD bands of *Eos.* **Figure S8.** Site-specific PCR with TE-flanking primer. **Figure S9.** A qPCR standard curve produced using primers for Traes_1BL_DD7D021A7 gene. **Figure S10.** Genome size (pg) of wild emmer wheat from 4 populations. (PDF 1237 kb)

## Abbreviations

AFLP: Amplified fragment length polymorphism
BLAST: Basic Local Alignment
LTR: Long terminal repeat
MITE: Inverted-repeat transposable element
NCBI: National center for biotechnology information
SINE: Small interspersed nuclear element
TD: Transposon display
TE: Transposable element
TRIM: Terminal inverted repeat in miniature
