# Supplementary material for: Transposable elements generate population-specific insertional patterns and allelic variation in genes of wild emmer wheat (Triticum turgidum ssp. dicoccoides)
Source: BMC Plant Biol. 2017 Oct 27;17:175. doi: 10.1186/s12870-017-1134-z (PMC5659041; doi:10.1186/s12870-017-1134-z)
Supplement: Supplementary file 1 — Primer sequences used for copy number variation analysis of TEs by real-time qPCR. Table S2. Adaptor and primer sequences used in Transposon Display (TD). Table S3. Primer sequences for site-specific PCR. Table S4. Primer sequences for site-specific PCR. (DOCX 28 kb) [file 12870_2017_1134_MOESM1_ESM.docx]

**Supplemental Table 1.** Primer sequences used for copy number variation analysis of TEs by real-time qPCR

| TE family^1^ | Forward primer | Reverse primer | Efficiency (%)^2^ |
| --- | --- | --- | --- |
| *Aison* | CTACCTCCGTCTCGGTGAATAAGT | AATCGTCAACCTAGAACTACGCG | 100 |
| *Eos* | GGGTTTGGAGGATAATAAATGCTC | ATGCTAGCCTCACCCATGCTT | 100 |
| *Fortuna* | GGATGCGGGAGTGAGAAGG | CTCCCATGCACAACAAGCC | 100 |
| *Minos* | GTAGTGCTTTCTCTATCCACGTGC | TTTACTTTCGCCGCAATCG | 91.4 |
| *Oleus* | CTCCCTCCGTTCACTATTATAAGATGT | CATACGGACTGAAATGAGTGAACAA | 100 |
| *Tantalos* | CTGGAAATGCTCATATTGTTTCCTAC | CGCATATTACCTTTGACTGAAGTTAAA | 100 |
| *Apollo* | GTCTGCCAAGTTTCATCAGGG | CGGCACCCACGAATGTTATT | 100 |
| *Balduin* | GAGAATGCTATGACGCAATGATG | TGGTACAGGTTTGCAGGCATAA | 92.4 |
| *Au* | AGCTGCTGCCTTGTGACCAT | GGGAAGGGTCCGACCACTT | 100 |
| *Veju* | TCGAGTCTCAAGGGTCGCA | TGGTCTGATGGAAGCGTGAA | 100 |
| *Fatima* | GCAGAGGAACTCACTGCGC | TCTTATCGAGTATCACATCGTCCCT | 97.4 |
| *Latidu* | CCGCTCATGGTGTTGGAGT | GAGCCGCATCGTGGACTG | 100 |
| *Latidu-LTR* | CGGAGTTGACGATCCCTCTC | GGCGATATCTGGTGATCAAGG | 93.7 |

^1^ Primers were previously described (Yaakov *et al*., 2012; Yaakov *et al.,* 2013)

^2^ Primers efficiency was tested by performing RT-qPCR reaction on serial dilutions of template cDNA mix and producing a standard curve. Efficiency = [(10^-1/y^) – 1] × 100% , where y is the standard curve slope.

**Supplemental Table** **2**. Adaptor and primer sequences used in Transposon Display (TD)

| Adaptors | *Mse*I adaptor 1 | TACTCAGGACTCAT |
| --- | --- | --- |
|  | *Mse*I adaptor 2 | GACGATGAGTCCTGAG |
| Pre-selective primer | *Mse*I preselective | GATGAGTCCTGAGTAAC |
| Selective primers^1^ | M-CTA | GATGAGTCCTGAGTAACTA |
|  | M-CTG | GATGAGTCCTGAGTAACTG |
|  | M-CTC | GATGAGTCCTGAGTAACTC |
| 5’ primers for MITEs^2^ | [^32^P] D-*Aison* | TTAAATCGTCAACCTAGAACTACGC |
|  | [^32^P] D-*Eos* | CAGGGGTGCTTGGAACTTTA |
|  | [^32^P] D-*Fortuna* | GTGGCCTTATATAGCTGCAAA |
|  | [^32^P] D-*Minos* | GTCAAAATTGAAGCACGTGGA |
|  | [^32^P] D-*Oleus* | CAAATCAGAAAGCTGGAACATC |
|  | [^32^P] D-*Tantalos* | TCTAGACAAACCTAGTATGCGGAGT |

^1^ M=*Mse*I.

^2^ Previously described by Yaakov *et al.* (2013). [^32^P] Primers labelled with ([γ-^32^P]-ATP). Each selective TD reaction included a primer combination TE-specific primer and one *Mse*I selective primer (M-CTA, M-CTG, or M-CTC)

**Supplemental Table 3.** Primer sequences for site-specific PCR

| Gene^1^ | TE insertion^2^ | Primer^3^ | Primer sequence |
| --- | --- | --- | --- |
| TRIUR3_24471 | *Aison,* 400 bp upstream | A1.11-F | TGCGTACCTTGATCCATTGTTAC |
|  |  | A1.11-R | AAAATAATATACCGCCGTCCAAG |
| TRIUR3_29094 | *Aison,* 195 bp upstream | A3t-F | TCGGTTCTATGCAATCTTCCAAG |
|  |  | A3t-R | TAAACAGGCCAAATCATCCGATC |
| Traes_2BS_2453C5E6B | *Aison,* intron 3 | A9t2-F | TGGGAGATAGTCTGTGTCACATT |
|  |  | A9t2-R | TCCCAATCAGCAGTTATCCCATA |
| Traes_7BL_2E24532BD | *Aison,* intron | A18t-F | GCCCTTGTTGACTTGCTTAGAT |
|  |  | A18t-R | TACGCACTAGGACAATGCTACT |
| FJ640556.1 | *Au,* intron 3 | AIP2.2-*Au*-F | TTGTGAGTTACCTTGAGCCTAGC |
|  |  | AIP2.2-*Au*-R | AGATTTGTGATAGCAGCACCAG |
| Traes_1BL_DD7D021A7 | *Au,* intron 5 | Q-F | TTACTGGGACCTTCCACACC |
|  |  | Q-R | GCCATCCATTTCCATTTCAG |
| TRIUR3_22200 | *Eos,* intron 4 | E3t-F | GGGCCTCCTCACTAGATAACAT |
|  |  | E3t-R | TTAGGAACGGAGGGAGTACTTG |
|  | *Thalos,* intron 1 | *Thalos*B-E3-i1-F | TCGGTCAGTGCATCATTTTCTC |
|  |  | *Thalos*B-E3-i1-R | CTCCTCCTACTGCTGCGATTT |
| Traes_6AL_80147B357 | *Eos,* exon | E15.1t-F | GCTCTGCTTTGTAGTTAATCGGT |
|  |  | E15.1t-R | CCGTGCATTTTCCACTCACTTAT |
| GU817319.1  locus tag - 2383A24.5 | *Hades,* 960 bp upstream | 2483A-F | CTCCGTCCCAAAAATGATGT |
|  |  | 2484A-R | GTATGTTCAAGGGTCGACCAAC |
| Traes_3AL_0D3EF0026 | *Minos,* 2 bp downstream | M9t-F | TATCCAGGTGCCATTTTGTGAGA |
|  |  | M9t-R | TACCGCACTTCCATTACCAGAAA |
| Traes_3AS_2755E639C | *Minos,* 22 bp upstream | M15Bt-F | TGCAGAACTGACATCATTAGAACT |
|  |  | M15Bt-R | CCTCCTGGTTTGTTGTCTGATT |
| Traes_7AL_0D3EF0026 | *Minos,* Exon 2 | M27.1t-F | GAAAGGAGGCATTGGACATTACA |
|  |  | M27.1t-R | TGTAATTGGAGCCAGTACTGAGT |
| Traes_3B_5DEF2D3F1 | *Tantalos,* Intron 3 | T1.17e3/e4-F | TCTTCGGGACAGTCTATTCTGAAG |
|  |  | T1.17e3/e4-R | CCCCGAGCTTTTCCTTCCTTAC |

^1^ Gene ID in EnsemblPlants database (<http://plants.ensembl.org/index.html>), or GenBank accession in NCBI database (<http://www.ncbi.nlm.nih.gov/>)

^2^ TE family and the exact insertion location within or close to the given gene, tested by ssPCR in this study

^3^ Primer name and direction: F – forward primer, R – reverse primer

**Supplemental Table 4.** Primer sequences used for gene expression analysis by real-time RT-PCR primers

| Gene^1^ | Forward primer | Reverse primer | Efficiency (%)^2^ |
| --- | --- | --- | --- |
| FJ640556.1 | GAGTTGAGGCCTGAGGAGATGA | TACCCTGCAGTGATGCTTCCA | 100 |
| Traes_3B_5DEF2D3F1 | GTGCTGCAAGAGACTATTGGCTATAT | AGTGCCATGTCCCAAGTATGG | 100 |
| Traes_1BL_DD7D021A7 | GAGCTAAAAGCGCTGCCTCTCT | TACAAGCCCCTTGAGCTCTGTG | 100 |
| Traes_3AL_0d3EF0026 | TGGCGGACCAGCTCACC | AATGCACGTCCCCATCCTT | 100 |
| Traes_7BL_2E24532BD | GATCAGGAACCATCGTGGTCTG | TTTCCTCTCCTGCCGGTAGTCT | 100 |
| Actin (reference gene) | GGCATGAGGAAGCGCATATC | ACTGGTATCGTTCTCGACTCTGG | 100 |

^1^ Gene ID in EnsemblPlants database (<http://plants.ensembl.org/index.html>), or GenBank accession in NCBI database (<http://www.ncbi.nlm.nih.gov/>)

^2^ Primers efficiency was tested by performing RT-qPCR reaction on serial dilutions of template cDNA mix and producing a standard curve. Efficiency = [(10^-1/y^) – 1] × 100% , where y is the standard curve slope.
