# Supplementary material for: Transposable elements generate population-specific insertional patterns and allelic variation in genes of wild emmer wheat (Triticum turgidum ssp. dicoccoides)
Source: BMC Plant Biol. 2017 Oct 27;17:175. doi: 10.1186/s12870-017-1134-z (PMC5659041; doi:10.1186/s12870-017-1134-z)
Supplement: Supplementary file 2 — Quantification of DNA elements in accessions of 5 wild emmer wheat population. Figure S2. Relative quantities of long TEs in 5 wild emmer wheat populations. Figure S3. Phylogenetic tree generated by hierarchical agglomerative clustering based on 99 TD bands of Aison. Figure S4. Phylogenetic tree generated by hierarchical agglomerative clustering on 70 TD bands of Fortuna. Figure S5. Phylogenetic tree generated by hierarchical agglomerative clustering based on 92 TD bands of Oleus. Figure S6. Phylogenetic tree generated by hierarchical agglomerative clustering based on 116 TD bands of Tantalos. Figure S7. Phylogenetic tree generated by hierarchical agglomerative clustering based on 92 TD bands of Eos. Figure S8. Site-specific PCR with TE-flanking primer. Figure S9. A qPCR standard curve produced using primers for Traes_1BL_DD7D021A7 gene. Figure S10. Genome size (pg) of wild emmer wheat from 4 populations. (PDF 1237 kb) [file 12870_2017_1134_MOESM2_ESM.pdf]

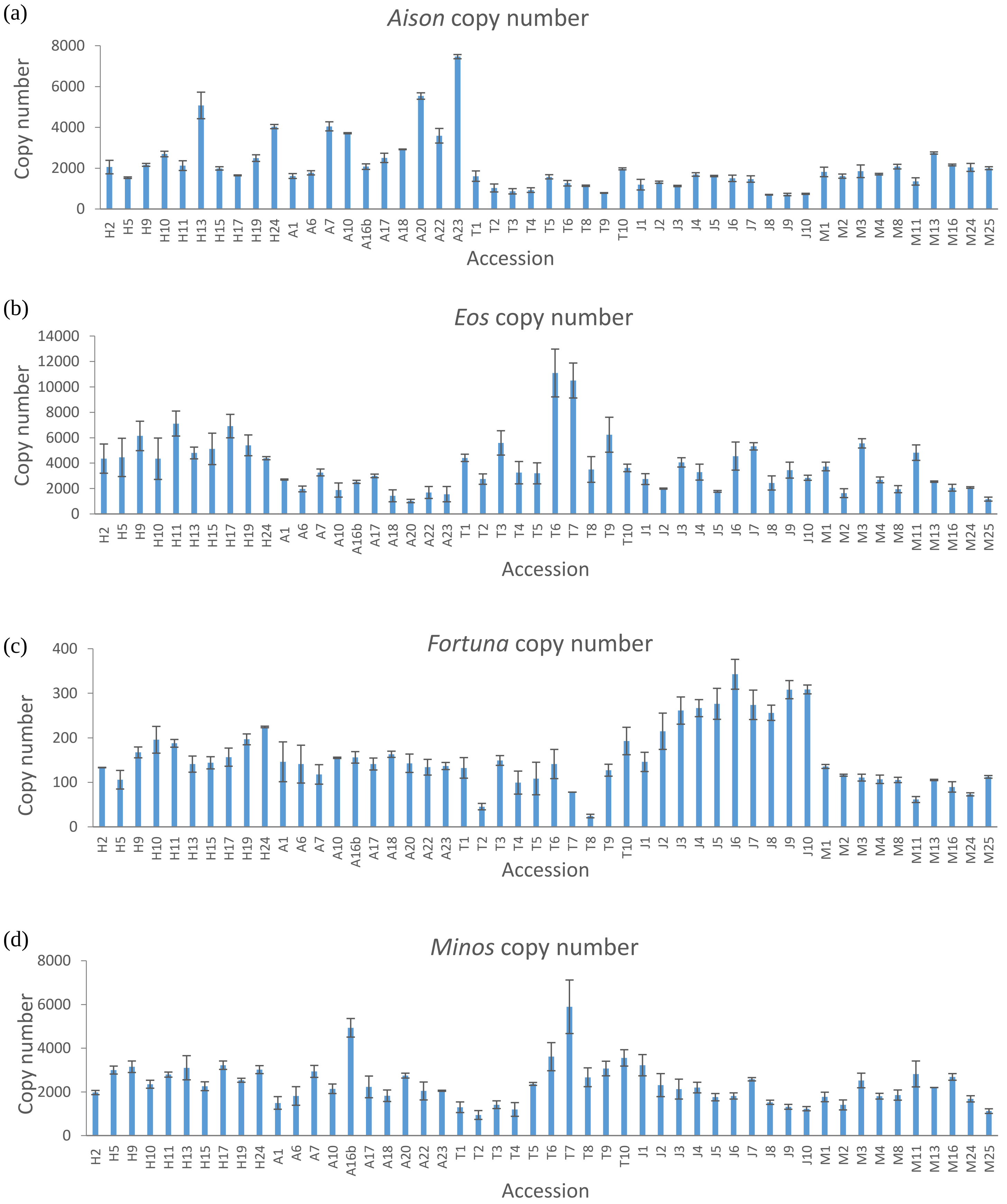

(a)
Aison copy number
Copy number
8000
6000
4000
2000
0
H2 H5 H9 H10 H11 H13 H15 H17 H19 H24 A1 A6 A7 A10 A16b A17 A18 A20 A22 A23 T1 T2 T3 T4 T5 T6 T8 T9 T10 J1 J2 J3 J4 J5 J6 J7 J8 J9 J10 M1 M2 M3 M4 M8 M11 M13 M16 M24 M25
Accession
(b)
Eos copy number
Copy number
14000
12000
10000
8000
6000
4000
2000
0
H2 H5 H9 H10 H11 H13 H15 H17 H19 H24 A1 A6 A7 A10 A16b A17 A18 A20 A22 A23 T1 T2 T3 T4 T5 T6 T7 T8 T9 T10 J1 J2 J3 J4 J5 J6 J7 J8 J9 J10 M1 M2 M3 M4 M8 M11 M13 M16 M24 M25
Accession
(c)
Fortuna copy number
Copy number
400
300
200
100
0
H2 H5 H9 H10 H11 H13 H15 H17 H19 H24 A1 A6 A7 A10 A16b A17 A18 A20 A22 A23 T1 T2 T3 T4 T5 T6 T7 T8 T9 T10 J1 J2 J3 J4 J5 J6 J7 J8 J9 J10 M1 M2 M3 M4 M8 M11 M13 M16 M24 M25
Accession
(d)
Minos copy number
Copy number
8000
6000
4000
2000
0
H2 H5 H9 H10 H11 H13 H15 H17 H19 H24 A1 A6 A7 A10 A16b A17 A18 A20 A22 A23 T1 T2 T3 T4 T5 T6 T7 T8 T9 T10 J1 J2 J3 J4 J5 J6 J7 J8 J9 J10 M1 M2 M3 M4 M8 M11 M13 M16 M24 M25
Accession

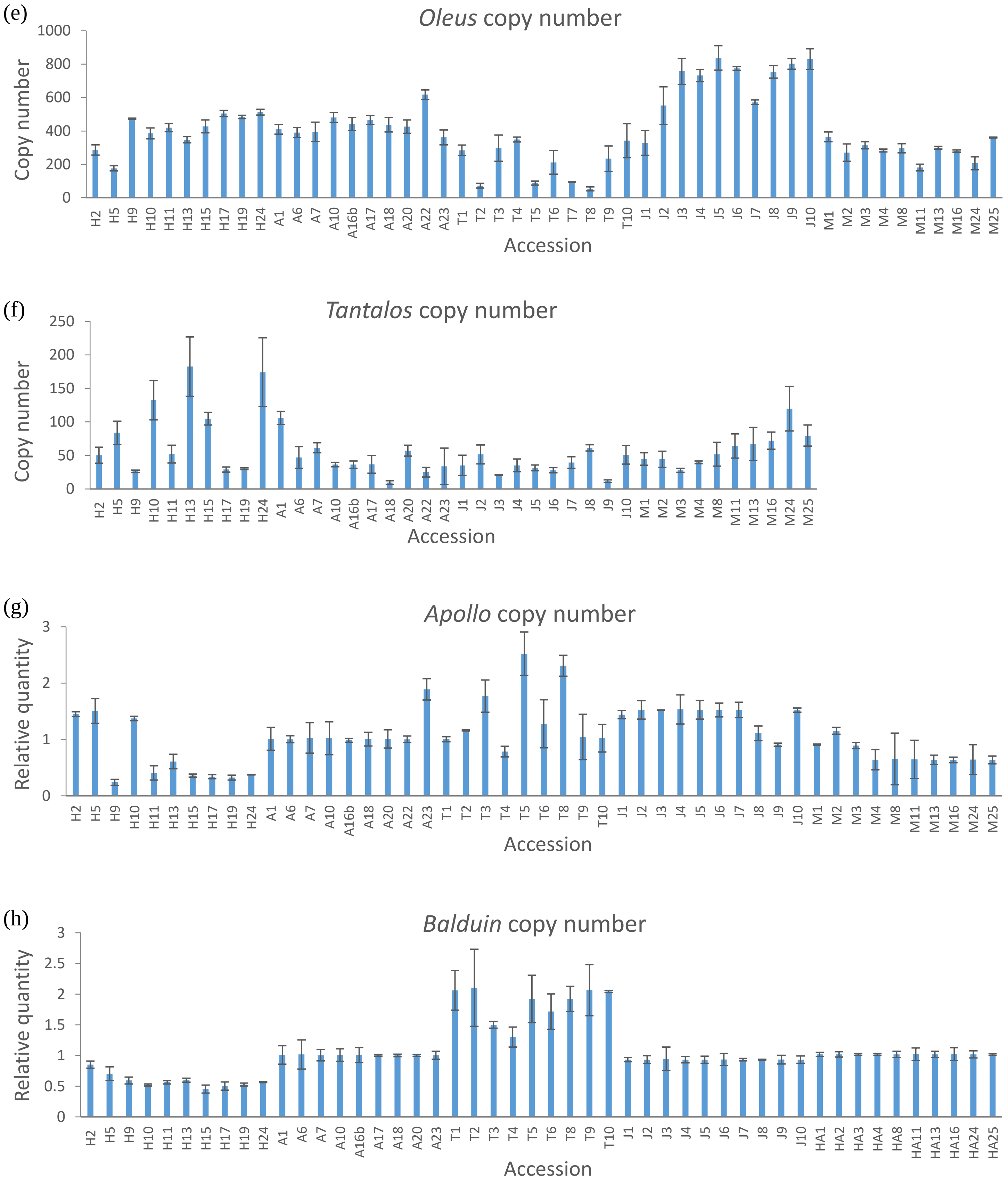

Supplemental Figure 1. Quantification of DNA elements in accessions of 5 wild emmer wheat populations (H - Mt. Hermon, A - Amiad, T - Tabgha, J - Jaba and M - Mt. Amasa). Error bars represent standard deviation of 3 technical replicates in qPCR reactions. **a-f** – MITEs: **a** - copy numbers of *Aison* in 46 accessions; **b** - copy numbers of *Eos* in 48 accessions; **c** - copy numbers of *Fortuna* in 50 accessions; **d** - copy numbers of *Minos* in 48 accessions; **e** - copy numbers of *Oleus* in 50; **f** - copy numbers of *Tantalos* in 40 accessions. **g, h** – long DNA elements: **g** - relative quantity of *Apollo* in 47 accessions; **h** - relative quantity of *Balduin* in 47 accessions.

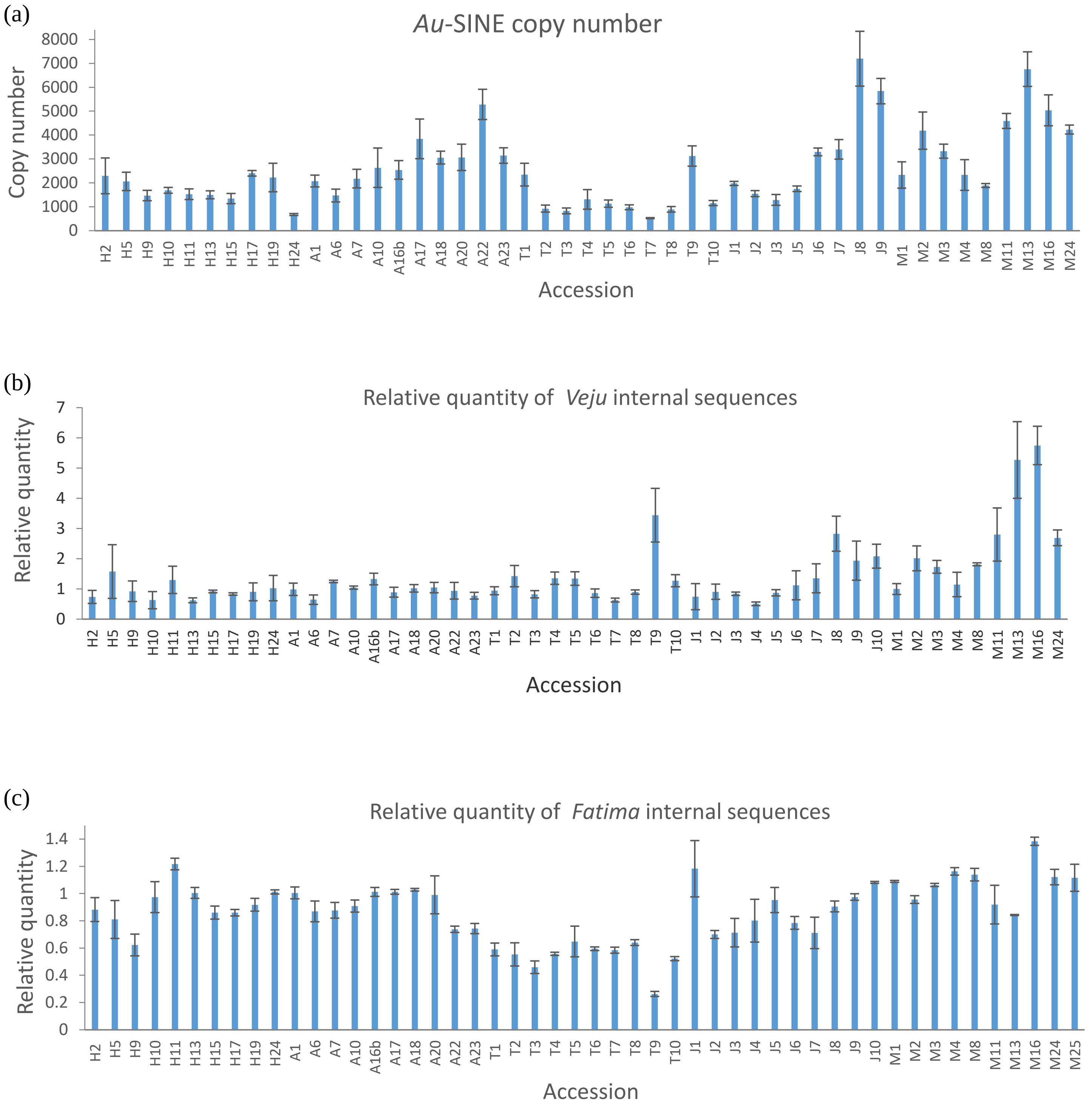

(a)
Au-SINE copy number
Copy number
8000 7000 6000 5000 4000 3000 2000 1000 0
H2 H5 H9 H10 H11 H13 H15 H17 H19 H24 A1 A6 A7 A10 A16b A17 A18 A20 A22 A23 T1 T2 T3 T4 T5 T6 T7 T8 T9 T10 J1 J2 J3 J5 J6 J7 J8 J9 M1 M2 M3 M4 M8 M11 M13 M16 M24
Accession
(b)
Relative quantity of Veju internal sequences
Relative quantity
7 6 5 4 3 2 1 0
H2 H5 H9 H10 H11 H13 H15 H17 H19 H24 A1 A6 A7 A10 A16b A17 A18 A20 A22 A23 T1 T2 T3 T4 T5 T6 T7 T8 T9 T10 J1 J2 J3 J4 J5 J6 J7 J8 J9 J10 M1 M2 M3 M4 M8 M11 M13 M16 M24
Accession
(c)
Relative quantity of Fatima internal sequences
Relative quantity
1.4 1.2 1 0.8 0.6 0.4 0.2 0
H2 H5 H9 H10 H11 H13 H15 H17 H19 H24 A1 A6 A7 A10 A16b A17 A18 A20 A22 A23 T1 T2 T3 T4 T5 T6 T7 T8 T9 T10 J1 J2 J3 J4 J5 J6 J7 J8 J9 J10 M1 M2 M3 M4 M8 M11 M13 M16 M24 M25
Accession

(d)

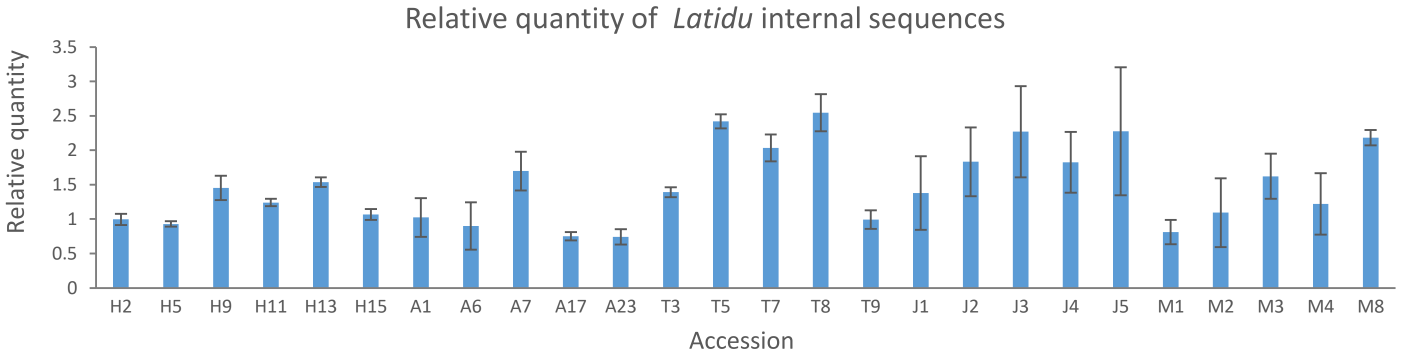

(e)

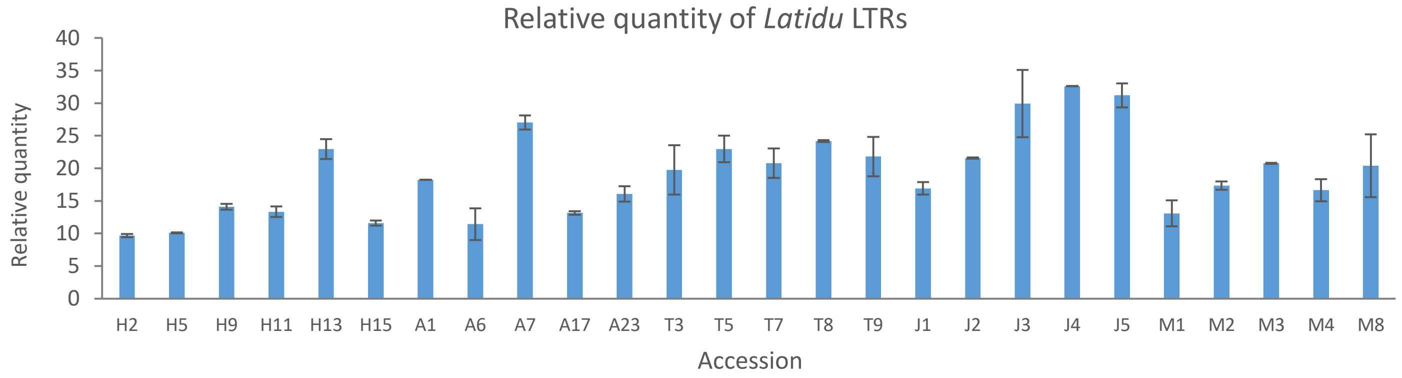

Supplemental Figure 2. Relative quantities of long TEs in 5 wild emmer wheat populations (H - Mt. Hermon, A - Amiad, T - Tabgha, J - Jaba and M - Mt. Amasa). Error bars represent standard deviation of 3 technical replicates in qPCR reactions. **a** - copy numbers of *Au* (retrotransposon) in 47 accessions; **b-e** – LTR retrotransposons: **b** - relative quantity of *Veju* in 49 accessions; **c** - relative quantity of *Fatima* in 50 accessions; **d, e** - relative quantity of *Latidu* in 26 accessions (**d** –based on internal sequence of *Latidu*, **e** – based on LTR sequence of *Latidu*).

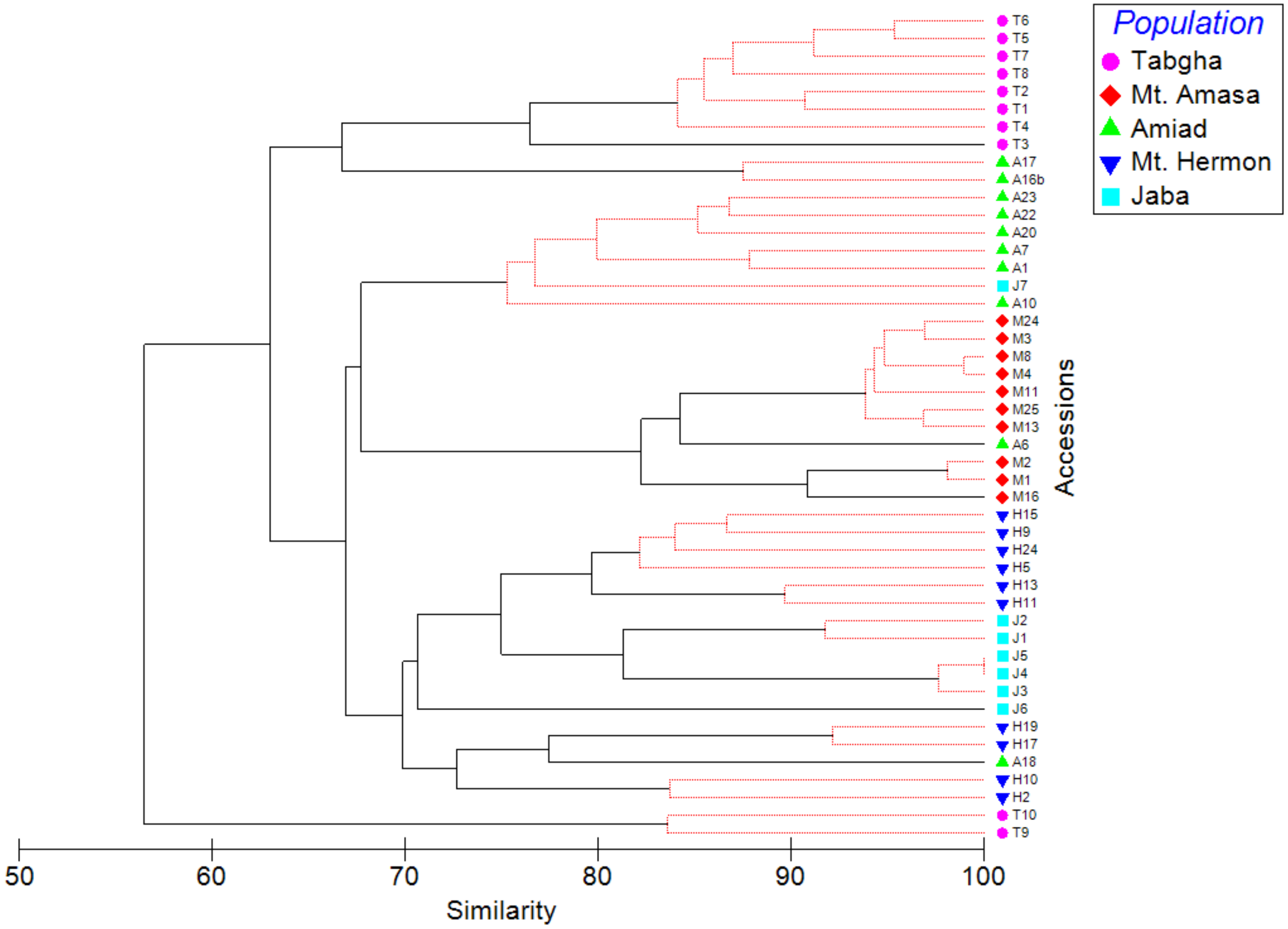

Supplemental Figure 3. Phylogenetic tree generated by hierarchical agglomerative clustering based on 99 TD bands of *Aison* in 44 accessions from 5 wild emmer wheat populations: Mt. Hermon, Amiad, Tabgha, Jaba and Mt. Amasa. The index (top right) shows the collection site of each accession. The black lines indicate significant separation, while red lines indicate insignificant separation. The level of similarity is indicated on the bottom.

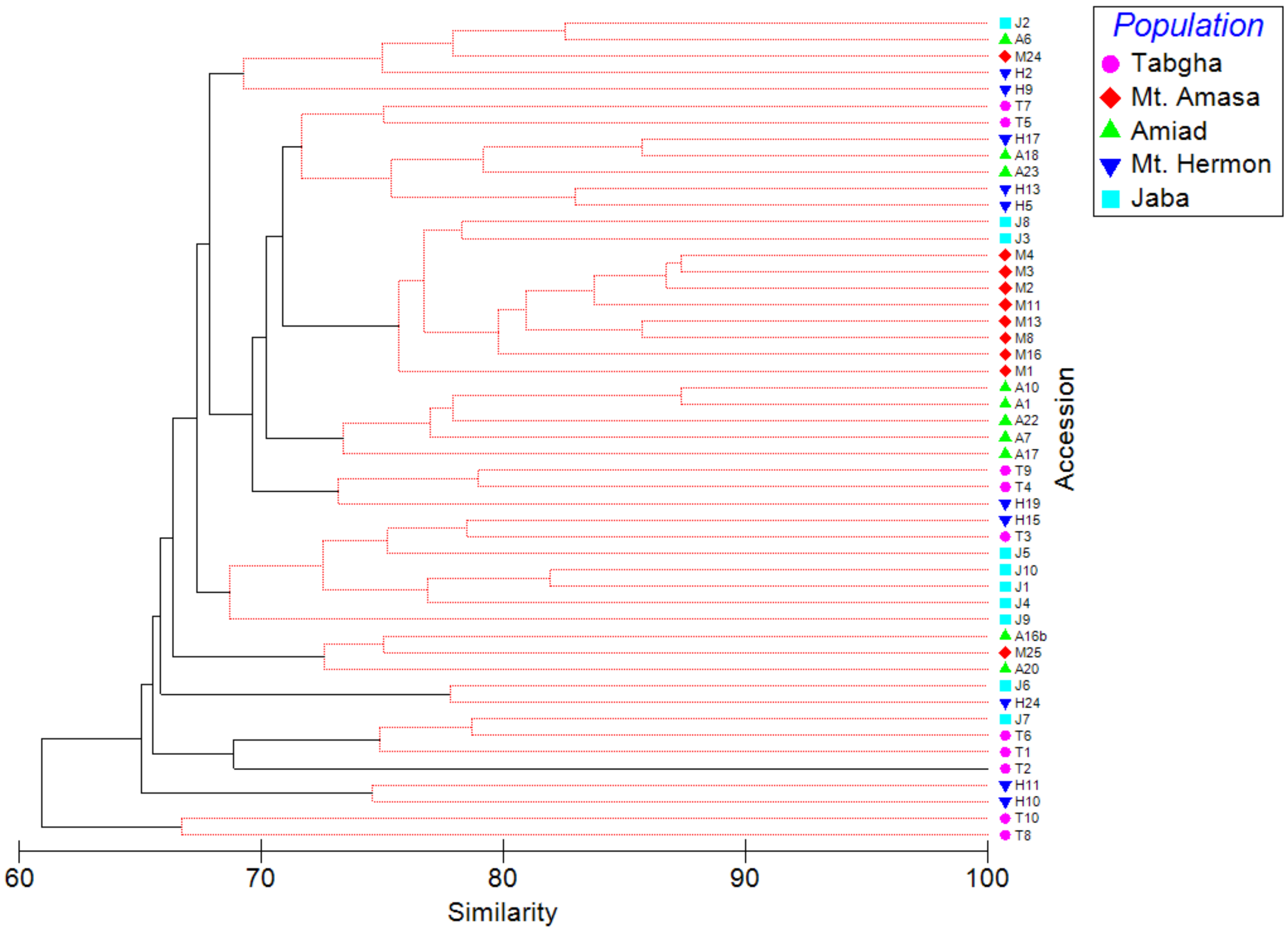

Supplemental Figure 4. Phylogenetic tree generated by hierarchical agglomerative clustering on 70 TD bands of *Fortuna* in 50 accessions from 5 wild emmer wheat populations: Mt. Hermon, Amiad, Tabgha, Jaba and Mt. Amasa. The index (top right) shows the collection site of each accession. The black lines indicate significant separation, while red lines indicate insignificant separation. The level of similarity is indicated on the bottom.

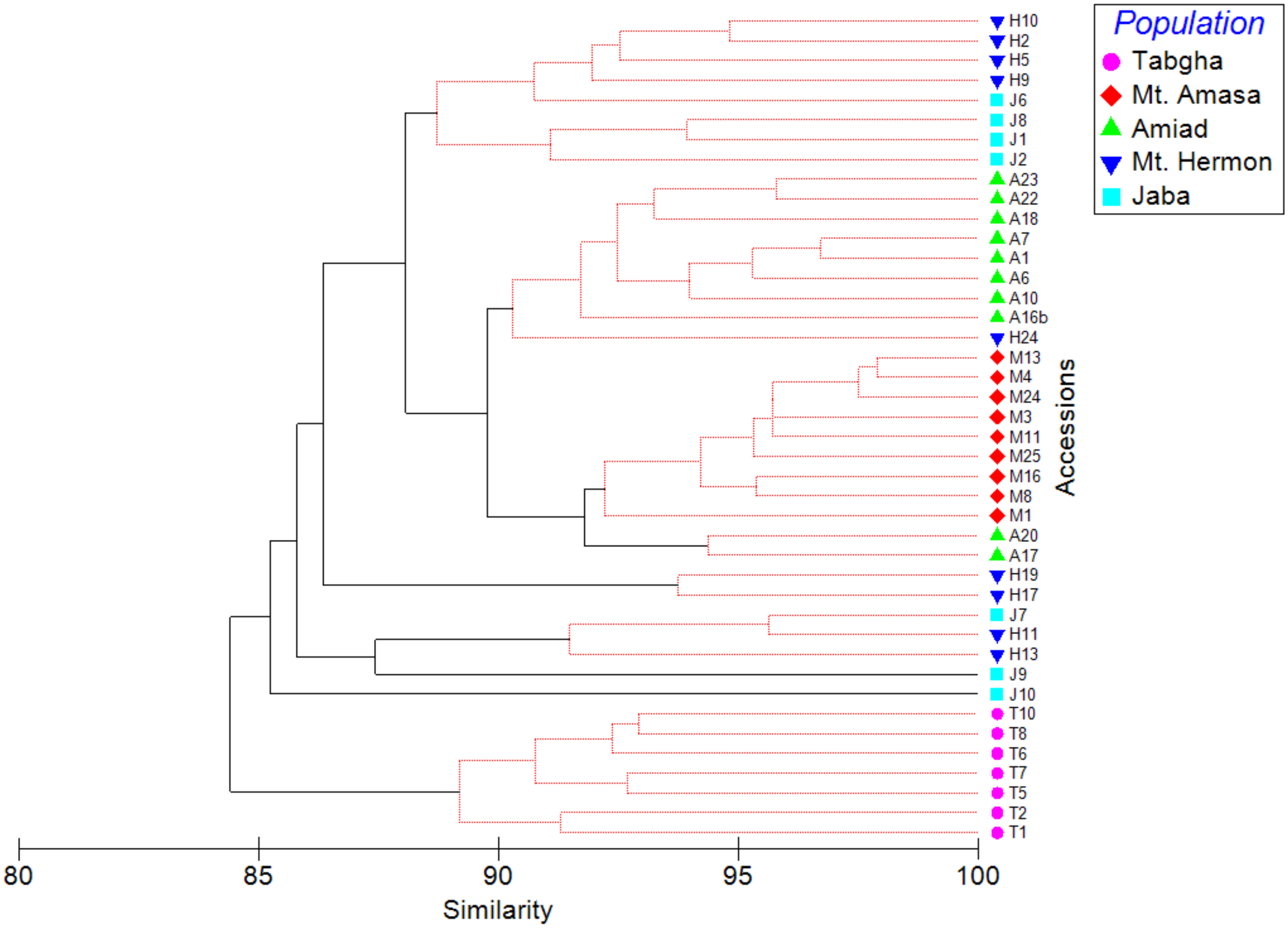

Supplemental Figure 5. Phylogenetic tree generated by hierarchical agglomerative clustering based on 92 TD bands of *Oleus* in 42 accessions from 5 wild emmer wheat populations: Mt. Hermon, Amiad, Tabgha, Jaba and Mt. Amasa. The index (top right) shows the collection site of each accession. The black lines indicate significant separation, while red lines indicate insignificant separation. The level of similarity is indicated on the bottom.

a.

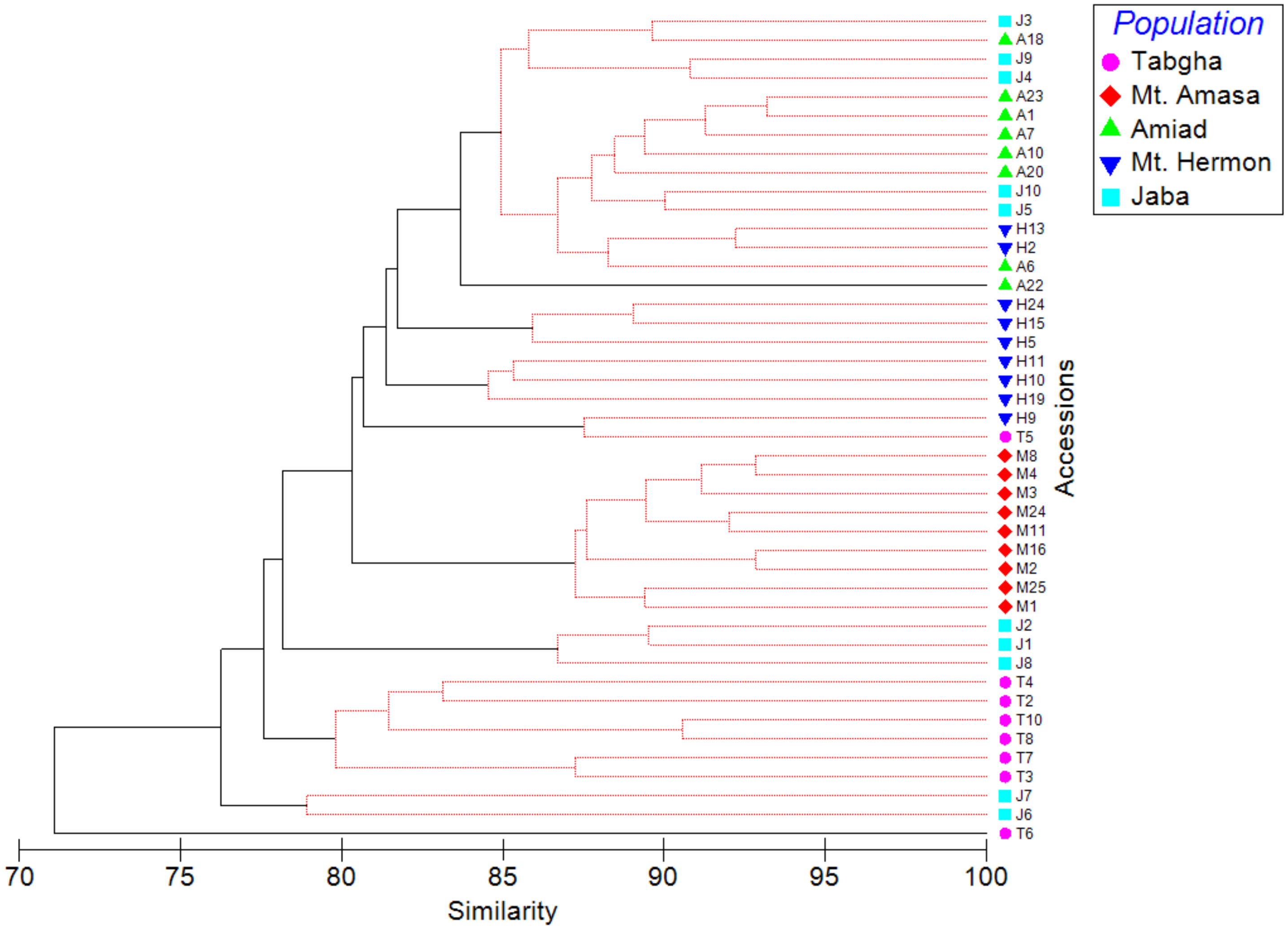

Supplemental Figure 6. Phylogenetic tree generated by hierarchical agglomerative clustering based on 116 TD bands of *Tantalos* in 44 accessions from 5 wild emmer wheat populations: Mt. Hermon, Amiad, Tabgha, Jaba and Mt. Amasa. The index (top right) shows the collection site of each accession. The black lines indicate significant separation, while red lines indicate insignificant separation. The level of similarity is indicated on the bottom.

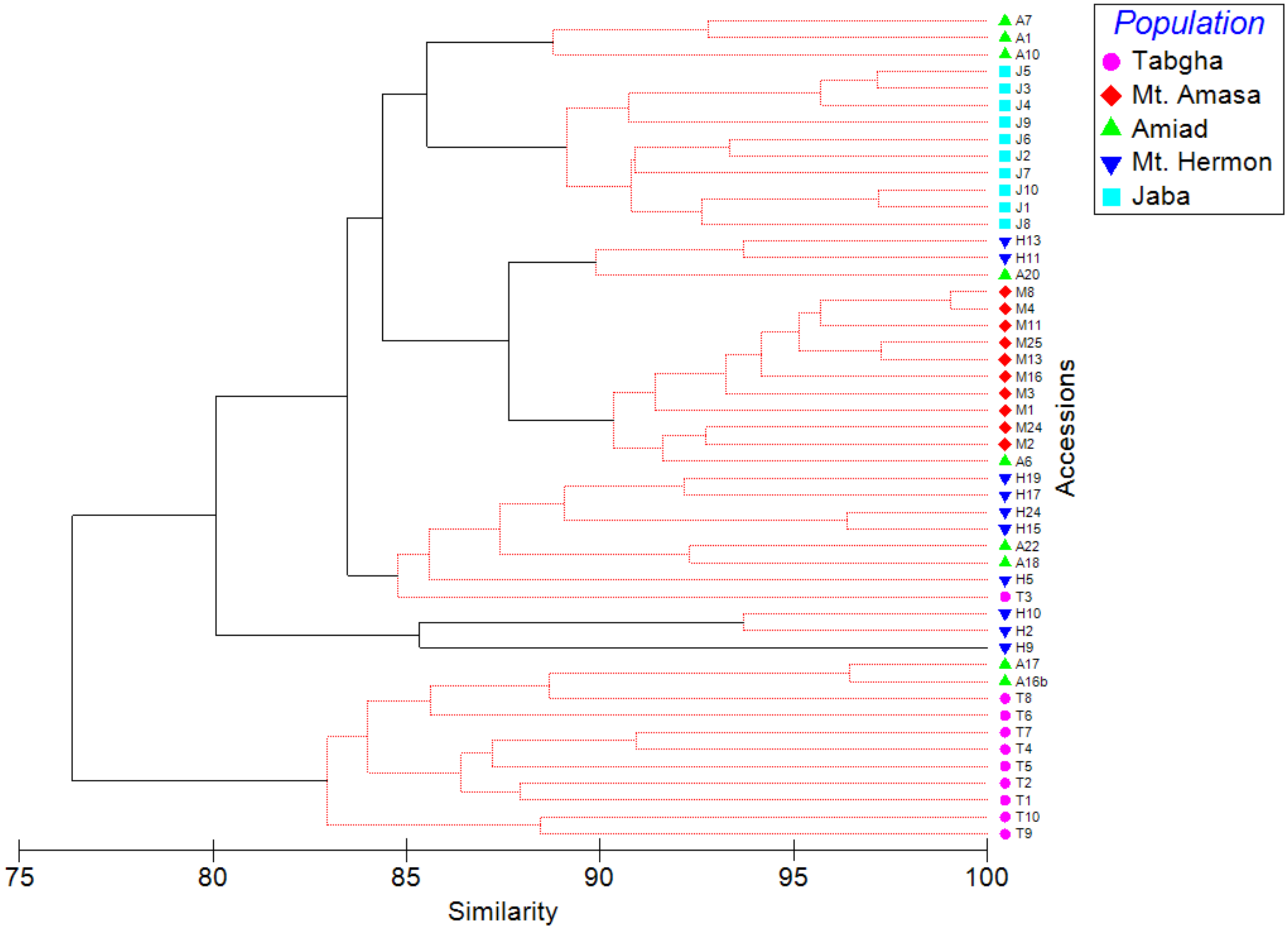

Supplemental Figure 7. Phylogenetic tree generated by hierarchical agglomerative clustering based on 92 TD bands of *Eos* in 49 accessions from 5 wild emmer wheat populations: Mt. Hermon, Amiad, Tabgha, Jaba and Mt. Amasa. The index (top right) shows the collection site of each accession. The black lines indicate significant separation, while red lines indicate insignificant separation. The level of similarity is indicated on the bottom.

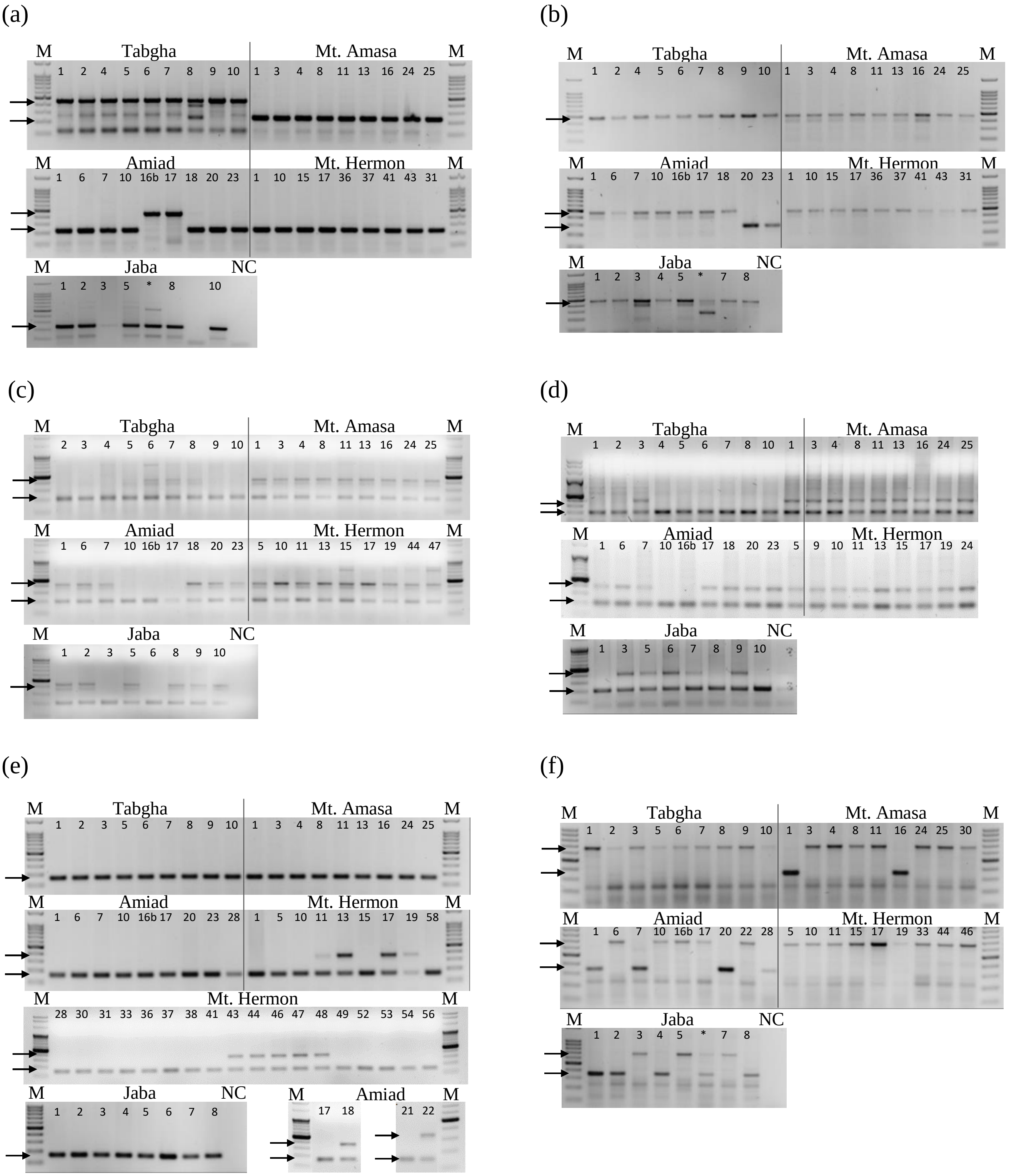

(a)
M Tabgha Mt. Amasa M
1 2 4 5 6 7 8 9 10 1 3 4 8 11 13 16 24 25
M Amiad Mt. Hermon M
1 6 7 10 16b 17 18 20 23 1 10 15 17 36 37 41 43 31
M Jaba NC
1 2 3 5 * 8 10
(b)
M Tabgha Mt. Amasa M
1 2 4 5 6 7 8 9 10 1 3 4 8 11 13 16 24 25
M Amiad Mt. Hermon M
1 6 7 10 16b 17 18 20 23 1 10 15 17 36 37 41 43 31
M Jaba NC
1 2 3 4 5 * 7 8
(c)
M Tabgha Mt. Amasa M
2 3 4 5 6 7 8 9 10 1 3 4 8 11 13 16 24 25
M Amiad Mt. Hermon M
1 6 7 10 16b 17 18 20 23 5 10 11 13 15 17 19 44 47
M Jaba NC
1 2 3 5 6 8 9 10
(d)
M Tabgha Mt. Amasa
1 2 3 4 5 6 7 8 10 1 3 4 8 11 13 16 24 25
M Amiad Mt. Hermon
1 6 7 10 16b 17 18 20 23 5 9 10 11 13 15 17 19 24
M Jaba NC
1 3 5 6 7 8 9 10
(e)
M Tabgha Mt. Amasa M
1 2 3 5 6 7 8 9 10 1 3 4 8 11 13 16 24 25
M Amiad Mt. Hermon M
1 6 7 10 16b 17 20 23 28 1 5 10 11 13 15 17 19 58
M Mt. Hermon M
28 30 31 33 36 37 38 41 43 44 46 47 48 49 52 53 54 56
M Jaba NC
1 2 3 4 5 6 7 8
M Amiad M
17 18 21 22
(f)
M Tabgha Mt. Amasa M
1 2 3 5 6 7 8 9 10 1 3 4 8 11 16 24 25 30
M Amiad Mt. Hermon M
1 6 7 10 16b 17 20 22 28 5 10 11 15 17 19 33 44 46
M Jaba NC
1 2 3 4 5 * 7 8

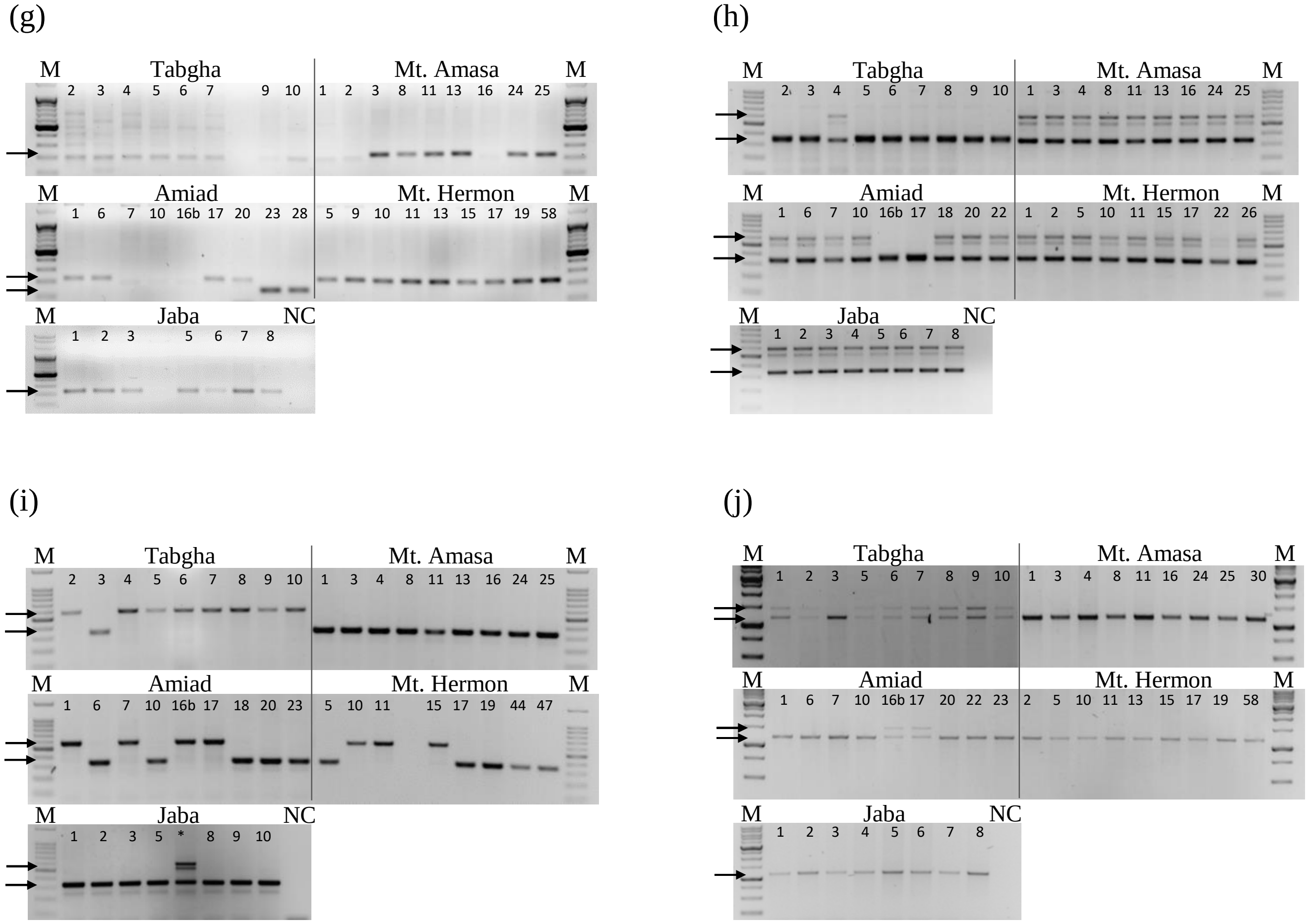

Supplemental Figure 8. Site-specific PCR with TE-flanking primers (**b, f, g, i**, – genome-specific primers; **a, c, d, e, h, j** – primers complementary to both A and B genomes) examining the presence or absence of miniature TE insertions within or close to 10 genes. Full and empty sites are indicated by arrows. M notes size marker. **a** - Insertion of *Aison* upstream to TRIUR3_29094 gene in all Tabgha (top left) and some Amiad (middle left) accessions, and absent from other populations. The upper band (453 bp) indicates full site, the lower band present in all accessions (234 bp) indicates empty site. **b** - Insertion of *Aison* in intron 3 of Traes_2BS_2453C5E6B gene, present in all accessions excepting two accessions of Amiad (middle left). The upper band (470 bp) indicates full site, the lower band (251 bp) indicates empty site. **c -** Insertion of *Aison* in intron of Traes_7BL_2E24532BD gene, present in all accessions of Mt. Amasa (top right) and Mt. Hermon (middle right) populations and in some accessions of Tabgha (top left), Amiad (middle left) and Jaba (bottom). The upper band (421 bp) indicates full site, the lower band present in all accessions (202 bp) indicates empty site. **d** - Insertion of *Au* in intron of FJ640556.1 (AIP2) gene, present in all accessions of Mt. Amasa (top right) and Mt. Hermon (middle right) populations and in some accessions of Tabgha (top left), Amiad (middle left) and Jaba (bottom). The upper band (409 bp) indicates full site, the lower band present in all accessions (207 bp) indicates empty site. **e** - Insertion of *Au* in intron of Traes_1BL_DD7D021A7 gene, present in some accessions of Amiad and Mt. Hermon and not in other populations. The upper band (366 bp) indicates full site, the lower band present in all accessions (164 bp) indicates empty site. **f** - Insertion of *Eos* in intron 4 of TRIUR3_22200 gene, present in all accessions of Tabgha (top left) and Mt. Hermon (bottom right), and in some accessions of Mt. Amasa (top right), Amiad (middle left) and Jaba (bottom). The upper band (679 bp) indicates full site, the lower band (339 bp) indicates empty site. **g** - Insertion of *Hades* upstream to 2383A24.5 gene, present in most accessions of all populations and absent from 2 accessions of Amiad (middle left). The upper band (228 bp) indicates full site, the lower band (142 bp) indicates empty site. **h** - Insertion of *Minos* downstream of Traes_3AS_94E185821 gene, present in all accessions of Mt. Amasa (top right), Mt. Hermon (middle right) and Jaba (bottom), and in some accession of Tabgha (top left) and Amiad (middle left). The upper band (593 bp) indicates full site, the lower band present in all accessions (356 bp) indicates empty site. **i** - Insertion of *Minos* in exon of Traes_7AL_0D3EF0026 gene, present in some accessions of Tabgha (top left), Amiad and Mt. Hermon (middle), and not in other populations. The upper band (582 bp) indicates full site, the lower band (345 bp) indicates empty site. **j** - Insertion of *Tantalos* in intron of Traes_3B_5DEF2D3F1 gene, present in most Tabgha (top left) and some Amiad (middle left) accessions and absent from other populations. The upper band (1444 bp) indicates full site, the lower band present in all accessions (1185 bp) indicates empty site.

* Lanes omitted from analysis due to possible sample contamination

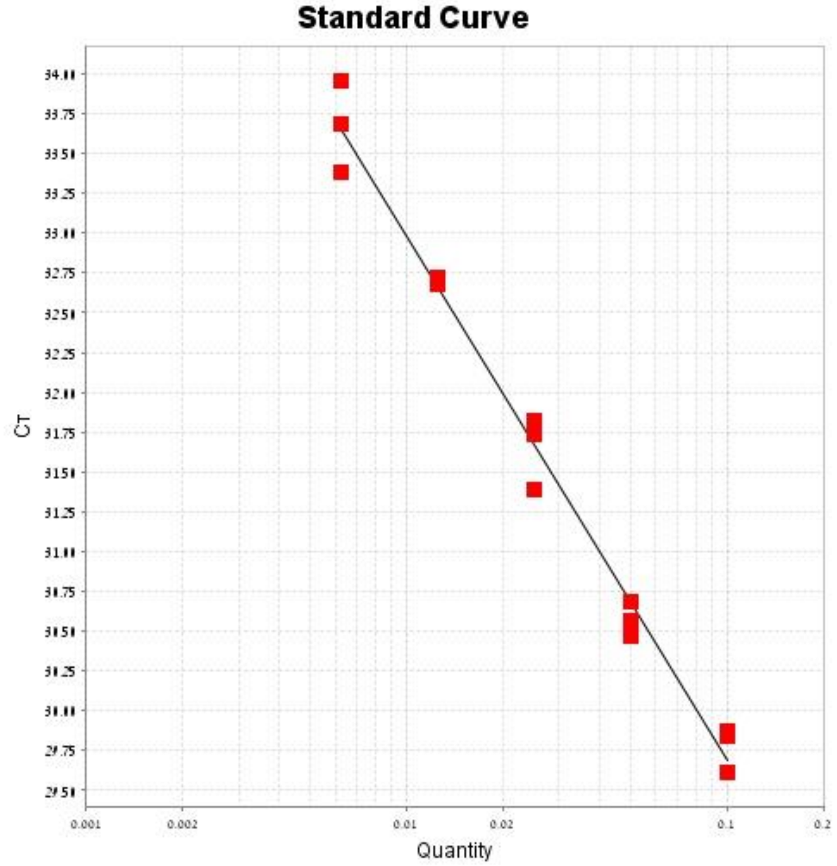

Supplemental Figure 9. A qPCR standard curve produced using primers for Traes_1BL_DD7D021A7 gene with a mix of cDNA templates. The X-axis represents the dilutions of cDNA. The efficiency of the primers, deduced from this standard curve, is indicated in Supplemental Table 4.

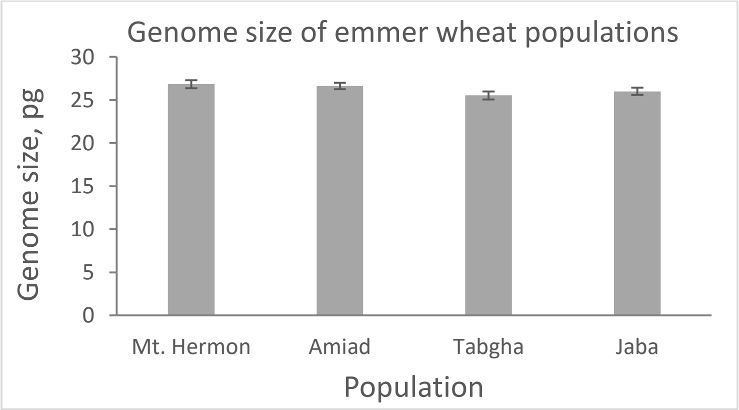

Supplemental Figure 10. Genome size (pg) of wild emmer wheat from 4 populations (Mt. Hermon, Amiad, Tabgha and Jaba), shown as average of 5 accessions of each population. Error bars indicate standard error.

Genome size of each accession was determined using Fluorescence-Activated Cell Sorting (FACS) technique. Nuclei were isolated from young leaves of T. dicoccoides and of tobacco (Nicotiana tabacum) used as a reference genome. Young leaves were chopped on ice with razor blade in nuclei isolation buffer. The fluid was filtered through 50 µm mesh and centrifuged for 5 min at 1000 × g at 4°C. The pellet was resuspended in 1.5 mL of nuclei isolation buffer, stained with Propidium Iodide dye and analyzed with flow cytometry using SY3200 cell sorter (i-Cyt). Genome size of wild emmer wheat accessions was calculated by normalization to values received for tobacco, with a known genome size of 10.3 pg (Johnson et al., 1999).
